# Age-dependent somatic expansion of the *ATXN3* CAG repeat in the blood and buccal swab DNA of individuals with spinocerebellar ataxia type 3/Machado-Joseph disease

**DOI:** 10.1007/s00439-024-02698-7

**Published:** 2024-10-08

**Authors:** Ahmed M. Sidky, Ana Rosa Vieira Melo, Teresa T. Kay, Mafalda Raposo, Manuela Lima, Darren G. Monckton

**Affiliations:** 1 School of Molecular Biosciences, College of Medical, Veterinary and Life Sciences, University of Glasgow, Glasgow, G12 8QQ UK; 2 Biochemistry Division, Chemistry Department, Faculty of Science, Minia University, Minia, 61519 Egypt; 3 Department of Neurology, University of Chicago, Chicago, IL 60637 USA; 4 Faculdade de Ciências e Tecnologia, Universidade dos Açores, Ponta Delgada, 9500-321 Portugal; 5 Unidade Multidisciplinar de Investigação Biomédica, Instituto de Ciências Biomédicas Abel Salazar, Universidade do Porto, Porto, Portugal; 6 Serviço de Genética Clínica, Hospital de D. Estefânia, Lisboa, Portugal; 7 Instituto de Biologia Molecular e Celular, Instituto de Investigação e Inovação em Saúde (i3S), Universidade do Porto, Porto, 4200-135 Portugal; 8 Present address: Surgery Brain Research Institute, J219, 5841 S. Maryland Avenue, Chicago, IL 60637 USA

## Abstract

Spinocerebellar ataxia type 3/Machado-Joseph disease (SCA3/MJD) is caused by the expansion of a genetically unstable polyglutamine-encoding CAG repeat in *ATXN3*. Longer alleles are generally associated with earlier onset and frequent intergenerational expansions mediate the anticipation observed in this disorder. Somatic expansion of the repeat has also been implicated in disease onset and slowing the rate of somatic expansion has been proposed as a therapeutic strategy. Here, we utilised high-throughput ultra-deep MiSeq amplicon sequencing to precisely define the number and sequence of the *ATXN3* repeat, the genotype of an adjacent single nucleotide variant and quantify somatic expansion in blood and buccal swab DNA of a cohort of individuals with SCA3 from the Azores islands (Portugal). We revealed systematic mis-sizing of the *ATXN3* repeat and high levels of inaccuracy of the traditional fragment length analysis that have important implications for attempts to identify modifiers of clinical and molecular phenotypes. Quantification of somatic expansion in blood DNA and multivariate regression revealed the expected effects of age at sampling and CAG repeat length, although the effect of repeat length was surprisingly modest with much stronger associations with age. We also observed an association of the downstream rs12895357 single nucleotide variant with the rate of somatic expansion, and a higher level of somatic expansion in buccal swab DNA compared to blood. These data suggest that the *ATXN3* locus in SCA3 patients in blood or buccal swab DNA might serve as a good biomarker for clinical trials testing suppressors of somatic expansion with peripheral exposure.

**Supplementary Information:**

The online version contains supplementary material available at 10.1007/s00439-024-02698-7.

## Introduction

Spinocerebellar ataxia type 3 (SCA3) (also known as Machado-Joseph disease (MJD)) is a rare autosomal dominantly inherited neurodegenerative disorder with a highly variable age at onset (Klockgether et al. 2019; Scott et al. 2020). SCA3-affected individuals show, in variable degrees, progressive gait ataxia, nystagmus, dysarthria, dystonia, distal muscle atrophy, ophthalmoplegia and fasciculations (Scott et al. 2020). Although the worldwide prevalence of SCA3 is ~ 1/100,000, SCA3 is nonetheless the most common SCA accounting for about 20 to 50% of SCA families worldwide (Klockgether et al. 2019). The prevalence of SCA3 is not equally distributed though, and SCA3 is more frequent in Portugal, particularly in the Azores islands, with an overall prevalence of ~ 1/2,500 and a remarkable 1/158 on Flores Island (de Araújo et al. 2016).

**Fig. 1:**
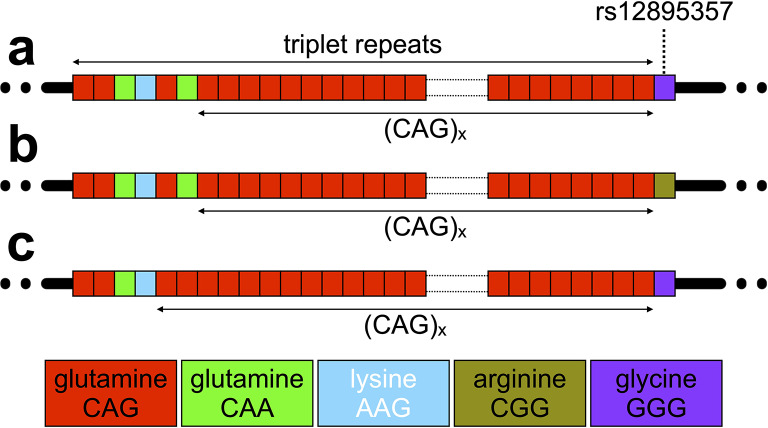
Allelic variation at the *ATXN3* triplet repeat locus. **a**) The schematic diagram shows the typical reference allele structure of the triplet repeat region in *ATXN3*, including the longest stretch of pure CAG repeats ((CAG)_x_). **b**) The non-reference arginine-encoding C-allele at the rs12895357 SNV. **c**) An atypical allele with loss of the CAA glutamine-encoding codon at repeat position six. The figure shows schematic representations of the alleles observed. Repeat codons are depicted: CAG glutamine codons as red boxes; CAA glutamine codons as green boxes; AAG lysine codons as sky blue boxes, CGG arginine codons as asparagus boxes; and GGG glycine codons as purple boxes

SCA3 is caused by the expansion of a polyglutamine-encoding CAG repeat in exon 10 of the *ATXN3* gene (NM_004993.6) (Kawaguchi et al. 1994). SCA3 is thus one of a group of disorders associated with the expansion of a glutamine-encoding CAG repeat, including the SCAs 1, 2, 7 and 17, DRPLA and Huntington disease (HD), that share a similar genetic mechanism and are assumed to also share a common toxic gain of function of the polyglutamine domain in the resultant protein (Bunting et al. 2022). The *ATXN3* polyglutamine-encoding CAG repeat varies from ~ 13 to ~ 44 in the general population (Lima et al. 2005; Gardiner et al. 2019), and from ~ 55 to ~ 90 in individuals affected by SCA3 (de Mattos et al. 2019; Akcimen et al. 2020). As with many other repeat expansion disorders (Depienne and Mandel 2021), measured repeat length is inversely correlated to age at onset accounting for ~ 55 to 62% of the variation in disease severity (de Mattos et al. 2019; Akcimen et al. 2020). The *ATXN3* repeat is complex with the reference sequence (NM_004993.6) incorporating two synonymous glutamine-encoding CAA variants at positions three and six, and a non-synonymous lysine-encoding AAG variant at position four, i.e., (CAG)_2_(CAA)_1_(AAG)_1_(CAG)_1_(CAA)_1_(CAG)_x_ (Kawaguchi et al. 1994; Igarashi et al. 1996) (Fig. 1). Direct Sanger sequencing of the *ATXN3* repeat in a small number of unaffected individuals (*n* = 36) and eleven patients from Japan (Kawaguchi et al. 1994) identified the presence of a sequence variant in which the CAA codon at position six is lost (see Fig. 1c). This loss-of-CAA variant is relatively common (~ 12%) in non-expanded alleles in the Japanese population, but appears less common (~ 2 to 5%) in other populations (Kawaguchi et al. 1994; Igarashi et al. 1996). As far as we are aware, neither the loss-of-CAA variant, or any other sequence variants have been identified within the *ATXN3* repeat in expanded alleles, albeit only very few expanded alleles appear to have been sequenced in their entirety. The repeat tract is followed immediately by a non-synonymous G/C single nucleotide variant (SNV) in a glycine codon (rs12895357, NM_004993.6(ATXN3):c.916G > C (p.Gly306Arg) (Fig. 1) (Kawaguchi et al. 1994; Igarashi et al. 1996). The non-reference arginine-encoding C-allele of rs12895357 is very common (Auton et al. 2015) and is the major allele on non-expanded chromosomes worldwide (Igarashi et al. 1996; Maciel et al. 1999). Likewise, most *ATXN3* expanded alleles are in phase with the C-allele, including all those reported in east Asian populations, but a subset of expanded alleles in populations of European ancestry are in phase with the less common glycine-encoding G-allele (Igarashi et al. 1996; Maciel et al. 1999).

As with many of the repeat expansion disorders (Depienne and Mandel 2021), expanded *ATXN3* alleles are intergenerationally unstable, with a bias toward expansion, explaining the anticipation observed in SCA3 and many related disorders (Maciel et al. 1995; Sasaki et al. 1995; Durr et al. 1996; Igarashi et al. 1996; Souza et al. 2016). Interestingly, the genotype of the rs12895357 SNV has been implicated in modulating the degree of intergenerational expansion: heterozygotes carrying the C-allele in phase with the expanded allele reportedly show higher levels of intergenerational instability in families and in sperm DNA directly, relative to homozygotes for either the C- or G-alleles (Igarashi et al. 1996; Takiyama et al. 1997; Maciel et al. 1999).

In addition to germline instability, again as with many repeat expansion disorders (Depienne and Mandel 2021), several small studies have revealed expanded *ATXN3* alleles to be somatically unstable in various tissues, including blood and brain (Cancel et al. 1995, 1998; Lopes-Cendes et al. 1996; Tanaka et al. 1996; Hashida et al. 1997; Maciel et al. 1997; Ito et al. 1998; Munoz et al. 2002).

The best practice guidelines for the molecular diagnosis of SCA3 is to PCR amplify across the whole repeat region using fluorescently tagged flanking primers, estimate fragment length by capillary gel electrophoresis and compare to known molecular weight standards to estimate the number of repeats assuming the canonical repeat structure (Sequeiros et al. 2010a, 2010b). Despite the fact that on average each extra *ATXN3* CAG repeat is predicted to be associated with an approximately 2- to 3-year decrease in SCA3 age at onset (de Mattos et al. 2019; Akcimen et al. 2020), the guidelines allow for an error margin of +/- 3 repeats (Sequeiros et al. 2010a, 2010b). Additionally, in a comparative study in which different protocols were used, only 17% of expanded alleles were sized the same between two independent laboratories, and ~ 6% differed by more than +/- 3 repeats (Ramos et al. 2016). In addition to frequent discrepancies in absolute length between laboratories, fragment length analysis reveals no information on the known polymorphic rs12895357 SNV, or potentially disease-modifying sequence variants within or flanking the repeat. Previously, we utilised high-throughput ultra-deep MiSeq amplicon sequencing to precisely genotype the *HTT* CAG repeat, revealing the critical role for repeat-sequence in driving disease severity, and accurately quantifying somatic expansion in the blood DNA of individuals with HD (Ciosi et al. 2019). It was our hypothesis that high throughput ultra-deep MiSeq amplicon sequencing of the *ATXN3* repeat might provide similar insights in individuals with SCA3.

## Materials and methods

### Cohort

A group of 142 molecularly confirmed SCA3 affected subjects from the Azorean SCA3 cohort (Lima et al. 2023), were recruited for this study. The Azorean SCA3 patients are derived from 32 extended families and are distributed on four of the nine Azorean islands (Lima et al. 1997). Both genealogical (Lima et al. 1998) and molecular evidence (Gaspar et al. 2001) confirm that more than one SCA3 mutation was introduced into the Azores, probably by settlers coming from the Portuguese mainland. Genomic blood DNA samples were used to determine the number of repeats in the *ATXN3* gene, define repeat structure, genotype the rs12895357 SNV, and quantify somatic expansions of all participants, in addition to a subset of 11 paired buccal swab DNA samples. Fragment length analysis of the *ATXN3* repeat number was performed using the MJD52/MJD70 PCR primers (Kawaguchi et al. 1994) as previously detailed (Bettencourt et al. 2008). A flow chart depicting which molecular and clinical data were available and used for each analysis is presented in Fig. S1.

### Illumina MiSeq sequencing of the *ATXN3* CAG repeat

The previously reported locus-specific primers MJD52/MJD70 (Kawaguchi et al. 1994) were modified to incorporate Illumina MiSeq adapter sequences, Nextera XT indexes and sequencing diversity increasing spacers, allowing the sequencing of multiplexed 384 amplicons as previously described for the *HTT* locus (Ciosi et al. 2018, 2019). The *ATXN3* CAG repeat was amplified by PCR in a total volume of 10 µl containing 10 ng of genomic DNA, 1 µM of forward and reverse MiSeq primers, 1X Custom PCR master mix (45 mM Tris-HCl, pH 8.8, 11 mM ammonium sulphate, 4.5 mM MgCl_2_, 4.4 µM EDTA, 1 mM dATP, 1 mM dCTP, 1 mM dGTP, 1 mM dTTP, and 113 µg ml^-1^ bovine serum albumin (Thermo Scientific, SM005), supplemented with 69 mM 2-mercaptoethanol)(Jeffreys et al. 1990) and 1 U of Taq polymerase (Sigma). PCR conditions were: an initial denaturation at 95°C for 5 min; followed by 25 cycles of denaturation (95°C for 1 min); annealing (56°C for 1 min); and extension (72°C for 1 min); and a final extension at 72°C for 5 min. Library clean up and fragment size selection were carried out using 0.8x AMPure XP magnetic beads (Beckman Coulter) to remove primer dimers (Ciosi et al. 2018). DNA library concentration was quantified by Qubit™ fluorometry using the Qubit™ dsDNA HS Assay Kit (Thermo Scientific). To check the amplicon sizes, verify the absence of primer dimers and the presence of amplicons of interest, and accurately measure the molar concentration of the sequencing library, capillary electrophoresis was carried out on the 2100 Bioanalyzer using the Agilent High sensitivity DNA kit (https://www.agilent.com). The DNA library was sequenced and demultiplexed at the College for Medical Veterinary and Life Sciences Shared Research Facilities, a service provider at the University of Glasgow (https://www.polyomics.gla.ac.uk/ngs_omics.html). Amplicons were sequenced on the Illumina MiSeq platform using the MiSeq reagent v3 kit (Illumina). As a sequencing control, PhiX DNA was added to the library at 5% molar concentration. The *ATXN3* library was sequenced with 400 nt forward reads and 200 nt reverse reads in a 600 nt-cycle run. Demultiplexing of the MiSeq reads was carried out according to the Illumina i5 and i7 indices from Nextera XT index kit v2, then the demultiplexed reads were exported in FASTQ file format for the subsequent bioinformatic analyses (Ciosi et al. 2018).

### Bioinformatic analyses of MiSeq amplicon libraries

Custom *ATXN3* reference sequences were generated using the RefGeneratr tool (https://github.com/helloabunai/RefGeneratr) with variable CAG repeat numbers and the two rs12895357 alleles; (CAG)_1 – 100_(G/C). Bioinformatic analyses of Illumina MiSeq sequencing reads was performed via the open-source Galaxy interface (Afgan et al. 2016), hosted by the University of Glasgow (http://heighliner.cvr.gla.ac.uk). Bioinformatic pipelines were set, and Galaxy workflows were created to allow the simultaneous analysis of the MiSeq sequenced *ATXN3* library. Briefly, the workflow included demultiplexing the reads using the locus specific primer and adaptor trimming Cutadapt tool (Martin 2011), followed by alignment of the trimmed MiSeq sequence reads to the *ATXN3* custom reference sequences using the Map-with-BWA_MEM tool (Li and Durbin 2009, 2010; Li et al. 2009). Following the alignment the generated BAM files which contain the mapped reads were then converted into SAM files by the BAM-to-SAM tool (Li et al. 2009). To remove reads with multiple alignments with the same quality score, additional filtering according to the mapping quality (MAPQ) score was applied so all alignments with MAPQ equal to zero were filtered out. Aligned filtered SAM files were downloaded and visualised using the Tablet genomic sequence viewer (Milne et al. 2013). The phased rs12895357 and repeat length haplotypes were determined by comparison of relative read depth from alignments to the (CAG)_1 – 100_(G/C) references (see Fig. S2). Only aligned reads with the correct phased rs12895357 genotype were used for downstream analyses.

### Genetic and statistical analyses

Statistical analyses were performed using R (version 4.3.2)(The R Core Team 2021) via RStudio (version 2023.12.1 + 402)(The RStudio Team 2016): single and multiple linear regression models were carried out using the lm() function; 95% confidence intervals (CI) for proportions using the prop.test() function; t-test using the t.test() function; F-test using the var.test() function. The somatic expansion ratio for the expanded *ATXN3* allele was calculated: (sum(N_+ 1_ to N_+ 10_)/N where N = phased read depth for the modal allele and N_+ 1_, N_+ 2_, etc., equal phased read depth observed at modal allele + 1 repeat, + 2 repeats etc., (Ciosi et al. 2018). In the multivariate regression analyses the somatic expansion ratio was log transformed to normalise the data. The somatic expansion index for the expanded *ATXN3* allele was calculated: (sum(N_+ 1_ * 1 + N_+ 2_ * 2 + N_+ 3_ * 3 …. to N_+ 10_)/(sum(N to N_+ 10_) where N = phased read depth for the modal allele, and N_+ 1_, N_+ 2_, etc., equal phased read depth observed at modal allele + 1 repeat, + 2 repeats etc., (Lee et al. 2010).

## Results

### High-throughput ultra-deep MiSeq sequencing and genotyping of the *ATXN3* repeat in individuals inheriting the SCA3 mutation

In order to assess sequence diversity at the *ATXN3* repeat in the SCA3 population, we adapted the high-throughput ultra-deep MiSeq sequencing assay we developed for analysing the HD-associated *HTT* CAG repeat (Ciosi et al. 2018; Ciosi et al. 2019) to the *ATXN3* locus. Briefly, the *ATXN3* repeat was PCR amplified using locus-specific primers incorporating MiSeq sequencing adaptors and spacers and sample-specific barcodes. Amplicons were pooled, purified, and sequenced at ultra-high depth using MiSeq with 400 nt forward reads and 200 nt reverse reads. We used this approach to attempt to sequence the *ATXN3* repeat from the blood DNA of 142 individuals inheriting SCA3-associated expansions belonging to the extensively studied Azorean cohort (Lima et al. 2023). In addition, we also sequenced the *ATXN3* repeat in paired buccal swab DNA samples in a subset of eleven individuals. Individuals were genotyped by aligning the forward reads to custom *ATXN3* reference sequences comprising a variable number of pure CAG repeats (0 to 100) and G or C at the site of the rs12895357 SNV which is located at the very first base after the CAG repeat region (Fig. 1). This allowed simple phasing of rs12895357 to the relevant allele in heterozygotes and provided a read length distribution for each participant (see Fig. S2).

Samples were sequenced at very high depth with an average read depth of ~ 24,000 aligned reads per sample. The read-depth for the shorter non-expanded alleles was generally very high (~ 17,500 aligned phased reads per sample), and in the vast majority of samples (*n* = 139) a clear modal allele was apparent (Fig. S3). However, there were two samples with a phased aligned read depth for the non-expanded allele of < < 50 reads, and, in these cases, it was not possible to confidently define a modal repeat length. As expected, the lower PCR efficiency associated with larger fragments resulted in a reduced read depth for the expanded allele (~ 6,500 aligned phased reads per sample). In cases with aligned phased read depths below ~ 300 reads, it was not possible to confidently define a modal repeat length. Thus, we were only able to define the modal repeat length for 119/142 individuals (see Fig. S4). In univariate analyses there was: a very strong positive correlation between total read depth for the expanded allele and the non-expanded allele (*r*^2^ = 0.622, *p* < 3 × 10^–16^, *n* = 141, Fig. S5a); no association between read depth of the non-expanded allele and allele length (*r*^2^ ~ 0, *p* = 0.687, *n* = 139, Fig. S5b); and a suggestive negative association between read depth of the expanded allele and allele length (*r*^2^ = 0.014, *p* = 0.103, *n* = 119, Fig. S5c). However, in a multivariate analysis there was a highly significant negative association between read depth of the expanded allele and expanded allele length (*r*^2^ = 0.632, *p* < 3 × 10^–16^, *p*_ExpandedAlleleLength_ = 5 × 10^− 7^, *n* = 119). These data suggest that the primary driver of reduced read depth in these samples was a lower concentration and/or lower quality of DNA, but that expanded allele length also reduced the read depth of the expanded allele.

**Fig. 2:**
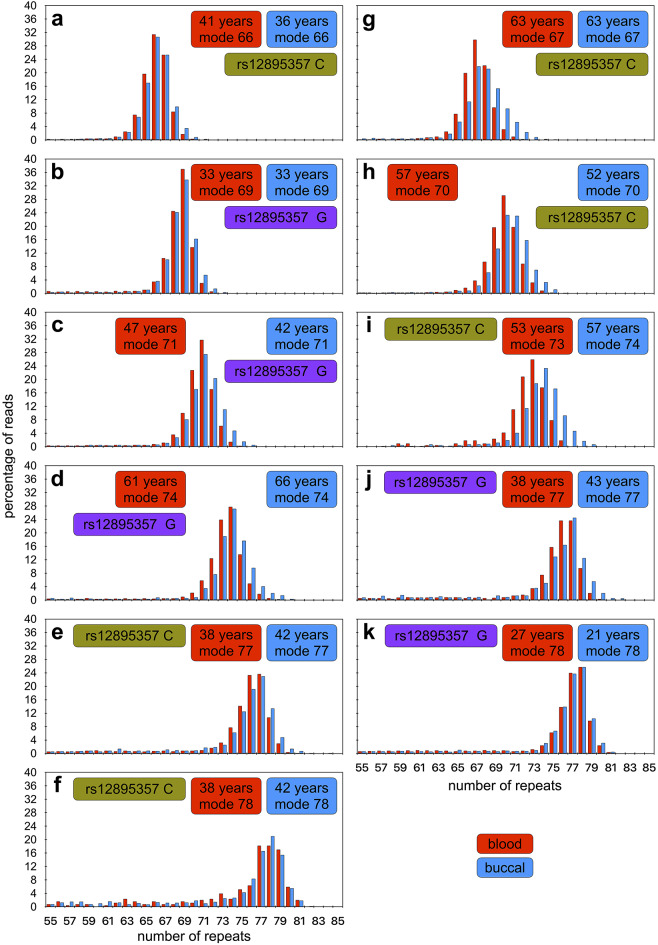
*ATXN3* triplet repeat read-length distributions for expanded alleles in blood and buccal swab DNA. **a** to **k**) The histograms show phased read-length distributions for the expanded *ATXN3* allele from both blood (red) and buccal swab (blue) DNA. MiSeq reads were aligned against references containing a variable number of CAG repeats and either the rs12895357 C-allele (asparagus) or G-allele (purple). For each participant the age at sampling and modal allele length observed for each tissue is indicated

Traditionally, the *ATXN3* repeat is genotyped by PCR amplification from blood DNA and the products resolved by fragment length analysis and the genotype defined as the number of repeats estimated in the modal peak(s) on fragment length analysis (Sequeiros et al. 2010a, 2010b). Most of the shorter non-expanded alleles (~ 74%, 98/137) were also genotyped as the same modal length using MiSeq and fragment length analysis (*r*^2^ = 0.989, *p* < 3 × 10^–16^, *n* = 137; estimation of repeat number by fragment length analysis was not available for two samples, Fig. S6a). However, ~ 24% (33/137) of these non-expanded alleles were estimated at one repeat smaller by fragment length analysis, two at two repeats smaller (~ 1.5%), and one at one repeat bigger (~ 0.7%). These differences were more pronounced for larger non-expanded alleles (*r*^2^ = 0.095, *p* = 0.0001, Fig. S6b) with ~ 94% (29/31) of 14 repeat alleles sized correctly, but only 65% (13/20) of 27 repeat alleles sized correctly, by fragment length analysis. The modal repeat length observed in the MiSeq read length distributions for the expanded alleles was the same in all the paired blood and buccal swab DNA samples (9/11) for which there was sufficient read depth (> 300 reads for the expanded allele in both samples, Fig. 2). Fragment length data was available for most of these individuals (98%, 117/119), but although there was a high correlation between the modal repeat lengths determined by MiSeq and fragment length analysis (*r*^2^ = 0.885, *p* < 3 × 10^–16^, Fig. S6c), all of the longer expanded alleles were sized shorter by fragment length analysis. The majority were estimated at either or two (36%, 42/117) or three (~ 35%, 41/117) repeats smaller by fragment length analysis, with a smaller number at one (~ 2.6%, 3/117), four (~ 10%, 12/117), five (~ 11%, 13/117), or even six repeats shorter (~ 5%, 6/117). These differences were not allele length-dependent (*r*^2^ ~ 0, *p* = 0.35, Fig. S6d).

All the expanded alleles (100%, 119/119), and most of the non-expanded alleles (~ 99.28%, 138/139), were revealed to present with the typical allele structure matching the genomic reference sequence, varying only in the number of pure (CAG)_x_ repeats and in their genotype at rs12895357. Only one non-expanded allele with loss of the second CAA glutamine-encoding repeat (see structure in Fig. 1c) was observed in an allele with a total repeat length of 22 repeats and a pure (CAG)_x_ tract of 18 repeats with the rs12895357 G-allele. After correcting for close family relationships, we observed allele frequencies of 69% G (33/48, 95% confidence interval (CI) = 54 to 81%) and 31% C (15/48, 95% CI = 19 to 46%) at rs12895357 on non-expanded alleles. These frequencies are consistent with those observed in European populations in the 1,000 genomes project (73:27%) (Auton et al. 2015) and as previously reported (Kawaguchi et al. 1994; Igarashi et al. 1996; Maciel et al. 1999). There were very high levels of linkage disequilibrium with all 14 and 23 repeat alleles in phase with rs12895357-G (Fig. S7a). Notably, we observed both rs12895357 alleles in phase with the expanded alleles: ~ 28% G (13/46, 95% CI = 16 to 44%); and ~ 72% C (33/46, 95% CI = 56 to 84%), consistent with at least two independent founder events for SCA3 in the Azores (Maciel et al. 1999; Martins et al. 2007). In all cases we observed the same phase between rs12895357 and expanded alleles within families, and clustering between islands (Fig. S7b/c). However, we did observe both rs12895357 alleles associated with expanded alleles in different families on one of the islands, consistent with at least two independent founders for SCA3 on São Miguel (Fig. S7b/c)(Maciel et al. 1999; Martins et al. 2007).

Interestingly, one individual presented without a detectable non-expanded allele in either the blood or buccal swab DNA samples by both fragment length analysis and MiSeq. The expanded allele(s) in this person were sequenced at very high depth (> 19,000 phased aligned reads) in both buccal swab and blood DNA, and both distributions looked very similar, with the same clear mode at 66 repeats (Fig. 2a) phased to the rs12895357-C allele. This individual’s father has a confirmed molecular diagnosis of SCA3, and their mother was reported by family members to also be affected. We thus assume that this individual inherited two expanded disease associated alleles of 66 and 67 repeats. Given the inability to partition somatic variants to the two alleles, this individual was excluded from the formal downstream somatic expansion and genotype-phenotype analyses (although see below).

### Somatic expansion of the *ATXN3* repeat

**Fig. 3:**
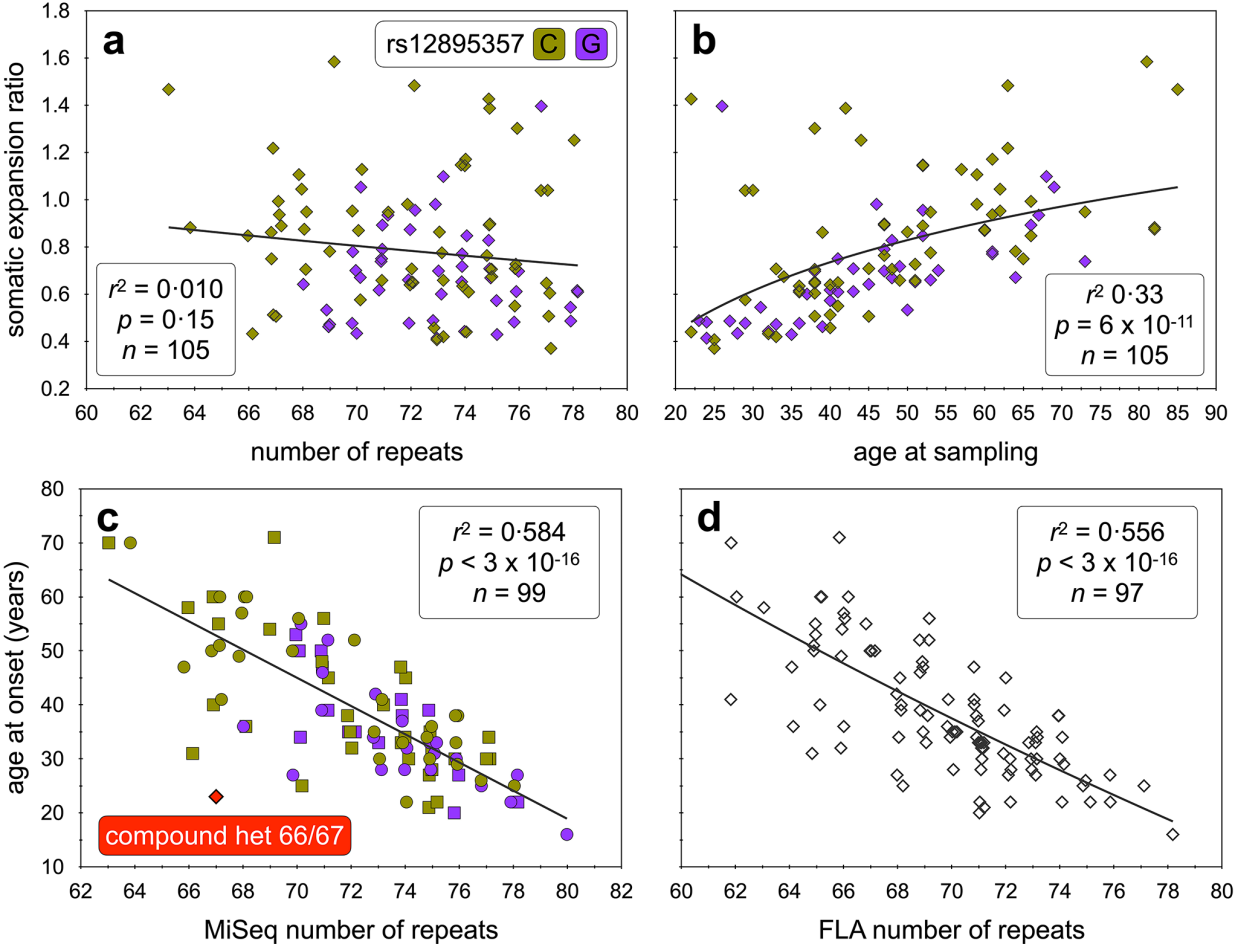
Modifiers of *ATXN3* triplet repeat blood DNA somatic expansion ratios and age at onset in SCA3. **a**) The scatterplot shows the ratio of somatic expansions of the expanded *ATXN3* alleles measured in blood DNA dependent on the number of *ATXN3* repeats. **b**) The scatterplot shows the ratio of somatic expansions of the expanded *ATXN3* alleles measured in blood DNA dependent on the age at sampling (years). **c**) The scatterplot shows the age at onset of SCA3 symptoms dependent on the number of *ATXN3* repeats as determined by MiSeq analysis in both females (circles) and males (squares). **d**) The scatterplot shows the age at onset of SCA3 symptoms dependent on the number of *ATXN3* repeats as determined by fragment length analysis. For each scatterplot the line of best fit (black line), adjusted coefficient of correlation squared (*r*^2^), *p*-value (*p*), and sample size (*n*) are indicated. For each participant rs12895357 genotype is also indicated: C-allele (asparagus) or G-allele (purple). Note, that to allow visualisation of overlapping points, random jitter (up to +/- 0.4 repeats) has been applied to the repeat numbers

It is well known that the PCR amplification of short tandem repeats typically results in the generation of a tail of Taq polymerase slippage products that are shorter than the input molecule(s) (see (Shinde et al. 2003)). Previously, by sequencing single DNA molecules containing the *HTT* CAG repeat, it was confirmed that PCR slippage generated a high proportion of artefactual products smaller than the progenitor allele, but only a very low proportion of PCR products, larger than the progenitor allele (Ciosi et al. 2019). These data suggest most expanded PCR products larger than the inherited progenitor allele in the bulk DNA sequencing data of the disease-causing alleles represent genuine somatic expansions (Ciosi et al. 2019). Inspection of the read length distributions for the non-expanded *ATXN3* alleles revealed a clear mode (Fig. S3a), representing the inherited progenitor allele (size = N), and the expected tail of shorter repeat length-dependent Taq polymerase slippage products (N_-1_ N_-2_, N_-3_, etc., Fig. S3b). The absence of products slightly larger than the inherited non-expanded alleles (N_+ 1_, N_+ 2_, N_+ 3_ etc.), suggest that, as expected, these alleles are somatically very stable. In contrast, the expanded alleles presented with much more diverse read-length distributions with many reads greater than the mode (Fig. 2, S4a–w). These diverse distributions likely reflect the combined contribution of PCR Taq polymerase slippage artefacts, and genuine somatic expansions. We assume that in the majority of cases: the modal allele represents the inherited progenitor allele (size = N); the tail of shorter products (N_-1_, N_-2_, N_-3_, N_-4_ etc.) represents primarily Taq polymerase slippage products; and that the tail of products larger than the mode (N_+ 1_, N_+ 2_, N_+ 3_, N_+ 4_ etc.), represent primarily somatic expansions (Fig. S5x). As we have previously done for similar analyses at the *HTT* locus (Ciosi et al. 2019), for those individuals with expanded allele read depth > 1,000 we thus quantified the degree of somatic expansion by calculating the somatic expansion ratio: (sum(N_+ 1_ to N_+ 10_)/N. We then performed a series of regression models to investigate the drivers of somatic expansion. Surprisingly, in univariate analyses there was no detectable main effect of repeat length on the somatic expansion ratio (*r*^2^ = 0.010, *p* = 0.15, *n* = 105, Fig. 3a), but there was a clear main effect of age at sampling (*r*^2^ = 0.33, *p* = 1 × 10^− 8^, *n* = 105, Fig. 3b). The lack of a detectable main effect for repeat length in a univariate analysis may reflect, at least in part, confounding effects of ascertainment bias. In sampled families there is a strong inverse correlation between age at sampling and repeat length (*r*^2^ = 0.29, *p* = 3 × 10^− 9^, *n* = 105, Fig. S8) i.e., individuals with larger repeats in the later generations of families present with an earlier age at onset and are sampled at an earlier age. Indeed, a multivariate model revealed highly significant effects for both age at sampling and repeat length, although there was no detectable interaction effect (*r*^2^ = 0.37, *p* = 2 × 10^–11^, *p*_*age*_ = 6 × 10^–12^, *p*_*repeat*_ = 0.008). Adding sex into the model revealed no detectable effect (*r*^2^ = 0.37, *p* = 1 × 10^–10^, *p*_*age*_ = 7 × 10^–12^, *p*_*repeat*_ = 0.009, *p*_*sex*_ = 0.98). However, the addition of the phased rs12895357 SNV yielded a substantive improvement in the model (*r*^2^ = 0.43, *p* = 8 × 10^–13^, *p*_*age*_ = 2 × 10^–12^, *p*_*repeat*_ = 0.002, *p*_*rs12895357*_ = 0.002). These data suggest that the rs12895357 C-allele is associated with higher rates of somatic expansion. Given the non-random distribution of the rs12895357 between islands, we also tested island origin as a cofactor, but this was not significant suggesting that island-specific *trans*-modifiers are not driving the rs12895357 association.

We also examined the degree of *ATXN3* somatic expansion in the DNA from eleven pairs of blood and buccal swab samples from the same individual (Fig. 2). Even though some buccal swab samples were taken at an earlier age then the blood DNA samples, all individuals showed a higher somatic expansion ratio in the buccal swab DNA than in the blood DNA. Indeed, combining the buccal swab and blood somatic expansion ratio data into the multivariate model derived for blood and adding tissue as an additional independent variable, confirmed the rate of expansion is indeed greater in buccal swab DNA relative to blood swab DNA (*r*^2^ = 0.50, *p* = 2 × 10^–16^, *p*_*age*_ = 1 × 10^–12^, *p*_*repeat*_ = 0.02, *p*_*rs12895357*_ = 0.001, *p*_*tissue*_ = 6 × 10^− 7^). Notably, individual-specific residual variation in buccal swab DNA was positively associated with individual-specific residual variation in blood DNA (*r*^2^ = 0.950, *p* = 0.0001, *n* = 7, Fig. S9).

In addition to utilising the somatic expansion ratio (Ciosi et al. 2019), we also calculated the somatic expansion index (Lee et al. 2010). The somatic expansion index also omits the tail of shorter Taq polymerase slippage products, but expansions are weighted such that it is broadly analogous to the average gain in CAG repeats at the time of sampling. Although the somatic expansion ratio and the somatic expansion index are highly correlated (*r*^2^ = 0.956, *p* < 3 × 10^–16^, Fig. S10), the somatic expansion index provides a simple route to estimating the rate of increase of CAG length with age. The rate of increase in CAG was estimated to vary from 0.008 to 0.037 CAG repeats year^-1^ with a mean of 0.013 CAG repeats year^-1^ in blood DNA (*n* = 105) and from 0.016 to 0.025 CAG repeats year^-1^ with a mean of 0.021 CAG repeats year^-1^ in buccal swab DNA (*n* = 9).

### Genotype-phenotype associations

As expected, the MiSeq modal allele length was inversely correlated with age at onset (*r*^2^ = 0.584, *p* < 3 × 10^–16^, *n* = 99, Fig. 3c). The modal allele length determined by fragment length analysis was slightly worse at predicting age at onset (*r*^2^ = 0.556, *p* < 3 × 10^–16^, *n* = 97, Fig. 3d). Neither sex, phased rs12895357 genotype, or residual variation in somatic expansion ratio in blood (corrected for repeat length, age and phased rs12895357 genotype) had any detectable association with age at onset (*r*^2^ = 0.575, *p* = 3 × 10^–15^, *p*_*repeat*_ = 3 × 10^–16^, *p*_*sex*_ = 0.492, *p*_*rs12895357*_ = 0.242, *p*_*ResidualSER*_ = 0.542, *n* = 99). Although not included in these formal genotype-phenotype analyses, it is apparent that the compound heterozygote inheriting two disease associated expansions of 66 and 67 repeats displays the largest deviation from the line of best fit with an age at onset ~ 30 years earlier than might be expected (Fig. 3c). Indeed, inclusion of this participant in the age at onset analyses using their largest allele (67 repeats) revealed participant zygosity to be a highly significant modifier (*r*^2^ = 0.587, *p* < 2 × 10^–16^, *p*_*zygosity*_ = 0.0003, *n* = 100) with an effect size of -29.8 years for compound heterozygosity, consistent with previous observations of more severe SCA3 symptoms in individuals homozygous for *ATXN3* expansions (Lysenko et al. 2010).

### Intergenerational transmissions

In this cohort we were able to successfully determine the difference in modal allele length in 32 intergenerational transmissions (17 maternal, 15 paternal) (Fig. S11). Intergenerational transmissions were significantly biased toward expansion (mean length change = + 0.94 repeats, *p* = 0.012), as expected. Not surprisingly, given the very small sample size, we were unable to detect significant expected effects of sex of parent or repeat length on the mean length of intergenerational transmissions (*p* > 0.05, data not shown). Similarly, we could detect no effect of parental rs12895357 genotype, either on the expanded allele chromosome, the non-disease associated allele, or parental zygosity (*p* > 0.05, data not shown). However, we did note that there appeared to be greater variance in male transmissions (variance = 7.4 repeats, range = -3 to + 7 repeats) relative to female transmissions (variance = 1.1 repeats, range = -1 to + 2 repeats) (Fig. S11b). This difference in variance between male and female transmission was highly significant (*F*-test, ratio of variances = 0.149, (95% CI = 0.051 to 0.421), *p* = 0.0005).

## Discussion

In this study we have used high-throughput ultra-deep MiSeq sequencing to successfully genotype the *ATXN3* CAG repeat in a large cohort of participants with SCA3 from the Azores. In addition to determining the modal repeat length, we were also able to determine the precise repeat structure, genotype and phase the rs12895357 SNV, and quantify the degree of somatic expansion for the expanded alleles. Although we successfully sequenced both *ATXN3* alleles for the vast majority of samples, the read depth for some samples was too low to confidently determine the modal allele or define the somatic expansion ratio. Given the very high correlation between read depth of the expanded and non-expanded alleles it would appear that the primary driver of these failures was DNA quality and/or DNA amount. Future studies should endeavour to ensure that the DNA used is of higher quality and/or the amplifiable DNA concentration more accurately determined. Notably, we revealed a systematic mis-sizing of the expanded allele with the traditional fragment length analysis consistently under-sizing the repeat length by two to three repeats (Fig. S6c/d). These systematic differences are not attributable to differences in the flanking primers used, as these were identical, but may be attributable to the known anomalous migration of CAG•CTG repeat containing DNA fragments in polyacrylamide gels (Chastain et al. 1995). More concerningly, some differences were as large as six repeats, and even in the best-case scenario assuming a systematic two repeat error, relative repeat sizes determined by fragment length analysis differed in 64% of cases (75/117). Whilst these differences have no impact on the ability to provide a yes/no diagnosis to patients and have only a modest impact on observed genotype-phenotype relationships, they may have a more substantive impact on research studies. For instance, the primary phenotype used in SCA3 genetic modifier studies is residual age at onset i.e., variation in age at onset not predicted by the repeat length of the expanded disease associated allele (de Mattos et al. 2019; Akcimen et al. 2020; Mergener et al. 2020; Raposo et al. 2022). Although such studies have started to reveal insights into pathways that may modulate SCA3, to date no genome-wide significant modifier variants have yet been defined. The two major factors mediating statistical power in genome-wide association studies are the robustness of the phenotype and the sample size. Whilst in general phenotypic robustness can be traded-off against sample size, in rare diseases such as SCA3, sample sizes are inevitably going to be rate limiting. Thus, there is a major impetus to increase the robustness of the phenotypes. Although residual variation in age at onset calculated using the MiSeq data and that calculated using the fragment length analysis data are highly correlated (*r*^2^ = 0.88, *n* = 97, *p* < 3 × 10^–16^), they are not the same and residual variation in age at onset calculated using MiSeq data should increase power in future genome-wide association studies. The genetic homogeneity of the Azorean population may have some advantages in identifying genetic modifiers of SCA3 (Raposo et al. 2022). Nonetheless, increasing sample size remains a major goal for genetic modifiers studies in SCA3 and the high-throughput nature of the MiSeq assay should greatly facilitate such studies in broader and more genetically diverse populations. In addition to effects on genotype-phenotype studies, and potentially even more substantively, mis-sizing repeat length by fragment length analysis has an even greater impact on sizing intergenerational transmissions with only 20% of intergenerational changes sized the same (*r*^2^ = 0.52, *n* = 31, *p* = 3 × 10^− 6^, Fig. S11e). Such errors in sizing intergenerational transmissions have obvious implications for their prognostic utility, and in efforts to identify their biological determinants and genetic modifiers.

In HD, the HTT polyglutamine domain is encoded by a mix of CAG and CAA codons that exist in a variety of combinations in both non-expanded, and expanded alleles. Critically, in HD the length of the pure CAG tract is a better predictor of disease severity than the length of the glutamine-encoding CAG/CAA tract (Ciosi et al. 2019; Genetic Modifiers of Huntington’s Disease Consortium et al. 2019; Wright et al. 2019). Theoretically, for an *ATXN3* allele that had lost both the CAA interruptions and the AAG interruption, the pure CAG tract would be mis-sized by six repeats by fragment length analysis (assuming accurate length determination). However, here we revealed only one non-expanded *ATXN3* allele that differed from the typical reference sequence with loss of the second CAA glutamine-encoding repeat (see structure in Fig. 1c). Fragment length analysis and repeat length determination by reference to the typical allele structure would underestimate the pure CAG repeat length by two repeats in this allele. The loss-of-CAA variant is relatively common (~ 12%) on non-expanded chromosomes in the Japanese population (Kawaguchi et al. 1994; Igarashi et al. 1996), but is clearly much less common in the Azorean population (~ 1%, 1/135 alleles), consistent with previous estimates (Igarashi et al. 1996). Likewise, previous analysis of expanded alleles in a sample of Azorean SCA3 patients using an allele-specific hybridisation assay did not detect this loss-of-CAA variant (Igarashi et al. 1996). Here we have confirmed this observation and revealed that no other *ATXN3* repeat sequence variants are present in expanded disease-associated alleles in the sampled Azorean SCA3 population. Thus, the relative importance of pure CAG length versus encoded-polyglutamine repeat length in SCA3 cannot be currently assessed. However, the availability of a high-throughput MiSeq assay should increase the feasibility of screening additional more ethnically diverse SCA3 cohorts for potentially very insightful repeat sequence variants.

**Fig. 4:**
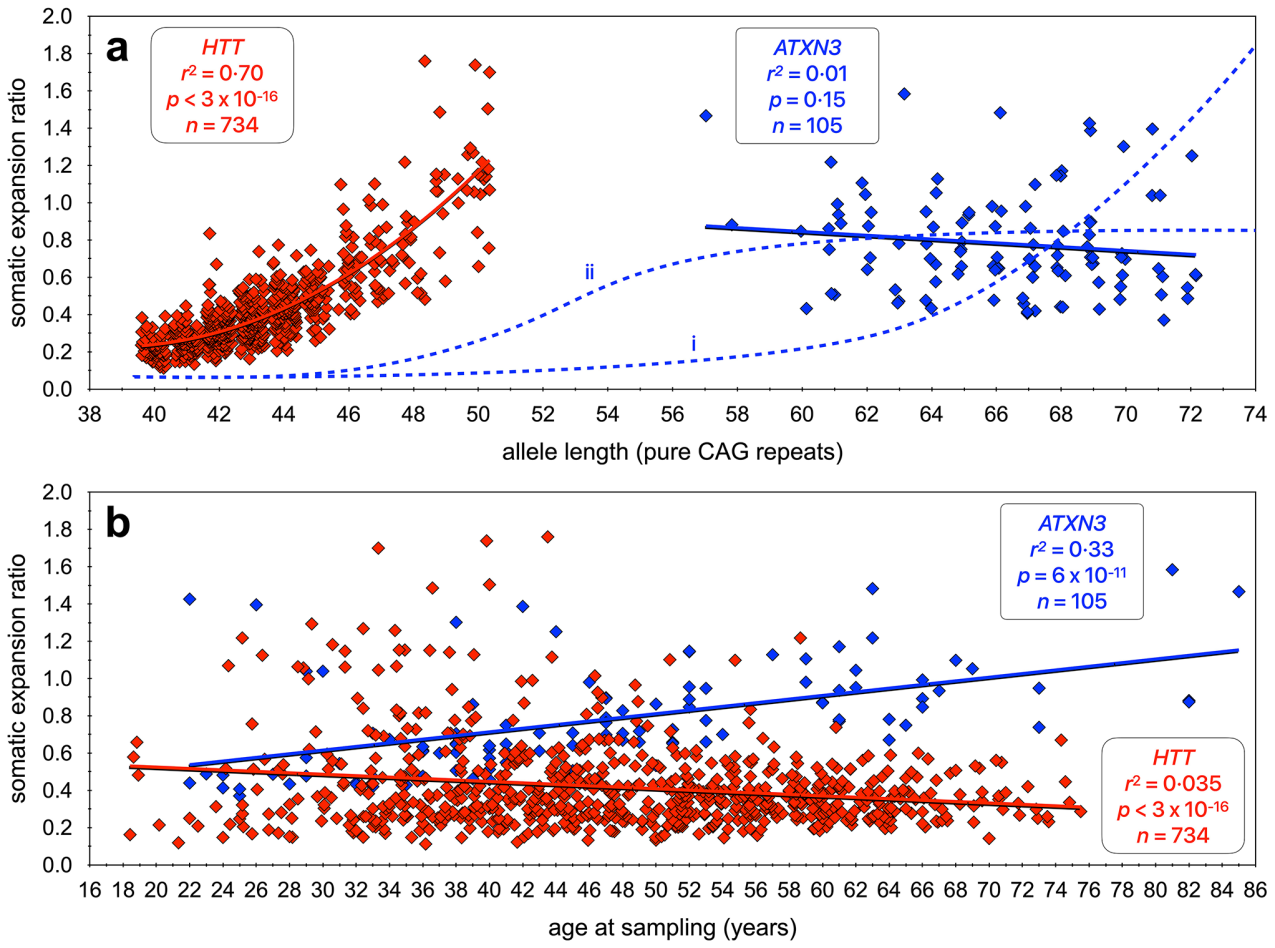
Contrast of the dynamics of somatic expansion of the *ATXN3* and *HTT* CAG repeats in the blood DNA. **a**) The scatterplot shows the ratio of somatic expansions of expanded *ATXN3* (blue points, this study) and *HTT* (red points, Ciosi et al. (2019) alleles measured in blood DNA dependent on the number of pure CAG repeats. **b**) The scatterplot shows the ratio of somatic expansions of expanded *ATXN3* (this study) and *HTT* (Ciosi et al. 2019) alleles measured in blood DNA dependent on the age at sampling (years). Note, that to allow visualisation of overlapping points, random jitter (up to +/- 0.4 repeats) has been applied to the number of repeats. The line of best fit (solid line), adjusted coefficient of correlation squared (*r*^2^), *p*-value (*p*), and sample size (*n*) for each univariate analysis are indicated for the *ATXN3* (blue) and *HTT* (red) datasets. Also illustrated in **a**) are hypothetical somatic expansion versus allele length relationships for *ATXN3* (blue dashed lines i and ii)

The greater prognostic value of pure CAG versus encoded-polyglutamine length in HD is assumed to be principally driven by the primary role of pure CAG length in influencing somatic expansion (Ciosi et al. 2019), and the importance of somatic expansion in modulating disease onset (Ciosi et al. 2019; Genetic Modifiers of Huntington’s Disease Consortium et al. 2019; Hong et al. 2021). Here, we utilised our MiSeq assay to quantify somatic repeat dynamics of the *ATXN3* repeat of the expanded allele in the blood DNA of the Azorean SCA3 cohort. Somewhat surprisingly, in clear contrast to the *HTT* CAG repeat (Ciosi et al. 2019), we did not detect an obvious main effect of repeat number on the somatic expansion ratio in a univariate analysis (Fig. 4a) as might have been expected (Fig. 4a, hypothetical line (i)). However, again in contrast to *HTT*, we did observe a very strong main positive effect of age at sampling (Fig. 4b). Nonetheless, as with *HTT*, in a multivariate model both age and repeat number were revealed as significant modifiers of the somatic expansion ratio. Whilst the lack of an obvious main positive effect for age at the *HTT* locus, or repeat length at the *ATXN3* locus, is likely at least partially attributable to the confounding effects of the age at sampling bias that characterises both disorders, the profound distinction between the two loci is noteworthy. One potential explanation is that most HD-associated *HTT* alleles are only just above the threshold at which somatic expansion in blood becomes detectable, and in a region where there is a clear exponential relationship between repeat length and somatic expansion (Fig. 4a). In contrast, the length of *ATXN3* alleles associated with SCA3 are much longer, and it is possible that the rate of change in somatic expansion with CAG repeat length is beyond the exponential phase and starts to plateau (Fig. 4a, hypothetical line (ii)). The lack of a major repeat length effect on somatic expansion has also been observed in a recent fragment length analysis study of longitudinal repeat dynamics in blood DNA of the expanded *ATXN3* CAG repeat in SCA3 (Kacher et al. 2024). In contrast to SCA3, and as with HD, this study also revealed clear allele length effects on somatic dynamics of the expanded *ATXN1* CAG in SCA1, the expanded *ATXN2* repeat in SCA2, and the expanded *ATXN7* CAG repeat in SCA7 (Kacher et al. 2024). Notably, expanded alleles for SCA1, SCA2, and SCA7 mostly fall within a similar 40 to 50 CAG repeat range as observed in HD, suggesting absolute length may be an important driver of the reduced length effect in SCA3. Consistently, germline transmission studies in SCA3 have also failed to reveal obvious parental allele length effects (Maciel et al. 1995; Durr et al. 1996; Martins et al. 2008; Souza et al. 2016) that are characteristic of SCA1, SCA2, SCA7, HD and most other repeat expansion disorders (Depienne and Mandel 2021).

Another interesting comparison is the obvious difference in absolute somatic expansion ratios between *HTT* and *ATXN3* relative to repeat length. Even after correcting for pure CAG repeat length, the *ATXN3* repeat is much more stable per CAG unit than the *HTT* locus (Fig. 4a). These data are consistent with major inter-CAG-repeat-locus *cis*-acting modifiers, as has been previously observed for germline repeat dynamics (Brock et al. 1999; Nestor and Monckton 2011) and has recently been observed in blood DNA for the expanded *ATXN1* CAG in SCA1, the expanded *ATXN2* repeat in SCA2, and the expanded *ATXN7* CAG repeat in SCA7 (Kacher et al. 2024). Indeed, the data here confirm the speculation that the *ATXN3* repeat is substantively more stable in the soma than the *HTT* repeat (Nestor and Monckton 2011). This observation appears to be replicated in the brain where, using fluorescence-activated nuclear sorting, it has recently been demonstrated that although unstable in medium spiny neurons, the *ATXN3* repeat in SCA3 donors was much more stable per repeat unit than the *HTT* repeat in HD donors (Mätlik et al. 2024). These data provide a potential explanation for the major difference in relative polyglutamine toxicity between the two loci as exemplified by the differences in the disease-associated range between HD (typically 40 to 60 polyglutamine-encoding repeats) and SCA3 (typically 60 to 80 polyglutamine-encoding repeats) i.e., that the intra-neuronal threshold for polyglutamine toxicity is higher than the inherited repeat length, and that individuals with SCA3 need to inherit a larger, but more slowly expanding, *ATXN3* allele to reach the cellular threshold at a similar age as an individual with HD inheriting a smaller, but more rapidly expanding, *HTT* allele (Nestor and Monckton 2011). Of course, differences in CAG-length dependent pathology between HD and SCA3 may also be mediated by differences in the protein context of the polyglutamine domain between HTT and ATXN3, which may be further modulated by CAG-length (in-)dependent effects on *HTT* and *ATXN3* splicing (Bettencourt et al. 2009; Harris et al. 2010; Sathasivam et al. 2013; Weishäupl et al. 2019; Hoschek et al. 2024).

A key role for somatic expansion in HD is also supported by the observation that residual variation in somatic expansion of the CAG repeat measured in blood (after correction for CAG and age-effects), is inversely correlated with variation in age at onset (Ciosi et al. 2019). This association is assumed to reflect the shared action of genetic modifiers that have similar effects on the rate of somatic expansion in affected neurons and haematopoietic stem cells. However, it should be noted that although highly significant, the effect size in HD was very modest (Ciosi et al. 2019) and this likely reflects the differential action of some modifiers between brain and blood (Ciosi et al. 2019; Lee et al. 2024). Here, we were not able to detect an association between residual variation in somatic expansion of the *ATXN3* CAG repeat measured in blood (after correction for CAG and age-effects) and variation in age at onset in our SCA3 data. However, it should be noted that the association in HD was detected in a much larger cohort (*n* = 734) (Ciosi et al. 2019). Thus, analysis of a much larger cohort will be required to rigorously test this hypothesis in SCA3. Nonetheless, albeit in a very small sample, we were able to demonstrate a striking correlation between individual-specific residual variation in blood and buccal swab DNA *ATXN3* somatic expansion ratios. Although, buccal swab DNA is likely to contain some contamination with white blood cells, the primary source of DNA is from the buccal cell epithelium suggesting a high degree of overlap in the action of genetic modifiers of somatic expansion between haematopoietic stem cells and buccal epithelial cells. Similarly, in myotonic dystrophy type 1 (DM1) it has been shown that individual-specific residual variation in *DMPK* CAG•CTG repeat somatic expansion (after correcting for allele length and age) was correlated across blood, skin and skeletal muscle DNA (Morales et al. 2023). Overall, these data suggest that genetic modifiers of somatic expansion may share more similar effects across peripheral tissues, and lower associations with brain. Data from a large cohort of post-mortem brains from SCA3 donors will be required to directly address this question.

Somatic expansion is now widely viewed as an important therapeutic target in HD, and likely in other related neurological disorders caused by the expansion of short tandem repeats, and efforts are afoot to identify drugs that might suppress somatic expansion (Benn et al. 2021). An important component of a clinical trial of any such entity will be to demonstrate that the drug does indeed suppress somatic expansion in vivo. Whilst ideally this would be demonstrated in the affected neurons, this is clearly not practical. Thus, at least for drugs with peripheral exposure, there is interest in identifying biomarkers in accessible peripheral tissues. Blood is the most obvious source of DNA for such analyses, but the levels of somatic expansion at the *HTT* locus in most individuals with HD inheriting less than 50 repeats is very modest, and detecting changes over short time periods is likely to be technically challenging. Although the rate of expansion of the *ATXN3* repeat is lower per repeat unit than at *HTT*, the average inherited repeat length in SCA3 patients is much higher and the average rate of expansion is higher (Fig. 4a). Despite the fact that SCA3 is much rarer than HD, the higher average rate of somatic expansion in SCA3, coupled with the lack of a major effect for repeat length, and the clear effect of age on somatic expansion of the *ATXN3* repeat in individuals with SCA3, suggest that SCA3 might present as an attractive disorder in which to establish proof-of-action in a clinical trial of a somatic expansion supressing drug with peripheral exposure. In addition, the demonstration here that the levels of somatic expansion were even higher in buccal swab DNA as opposed to blood cells, suggest that *ATXN3* expansion in buccal cells may be an even more powerful biomarker. However, the average rate of change observed in blood (0.013 CAG repeats year^-1^) or buccal swab DNA (0.021 CAG repeats year^-1^) seem rather low. Nevertheless, the utility of the *ATXN3* repeat in SCA3 as a potential biomarker, is supported by the recent demonstration of detectable longitudinal increases in repeat length in blood DNA over an average 8 year period (Kacher et al. 2024). These data were obtained using semi-quantitative fragment length analysis (Kacher et al. 2024). Ultra-deep high-throughput MiSeq sequencing offers the potential to dramatically increase sensitivity of the assay and reduce the time period over which longitudinal changes can be assessed.

Interestingly, we revealed here an apparent association of the rs12895357 C-allele with higher rates of somatic expansion in blood DNA. These data are consistent with the critical role of *cis*-acting elements in modulating inter-locus differences in expansion rates (Brock et al. 1999; Nestor and Monckton 2011), and suggest that naturally occurring *cis*-acting variants may also modulate intra-locus differences. These data may also relate to the previous observation that this variant may also be associated with altered rates of intergenerational expansion (Igarashi et al. 1996; Takiyama et al. 1997; Maciel et al. 1999). However, a simple *cis*-modifier effect of rs12895357 genotype on the intergenerational dynamics of the linked *ATXN3* repeat allele was not observed. Rather, rs12895357 heterozygotes carrying the C-allele phased with the expanded repeat appeared to have higher rates of instability compared to C/C or G/G rs12895357 homozygotes (Igarashi et al. 1996; Takiyama et al. 1997; Maciel et al. 1999). Such an effect could conceivably be mediated by inter-chromosomal interactions during meiosis, although this effect was not replicated in an independent analysis of sperm DNA analysis (Grewal et al. 1999). We observed no effect of rs12895357 on the intergenerational dynamics assessed in this study, but our sample size was very small (32 transmissions). Further larger studies will be required to better evaluate the effects of rs12895357 on both somatic and germinal *ATXN3* repeat dynamics.

As with previous observations (Lysenko et al. 2010), the individual identified here as homozygous for *ATXN3* expansions had a much earlier age at onset than expected. These data contrast with the situation in HD where homozygotes are not more severely affected and the age at onset is predicted by the length of the largest allele (Lee et al. 2012; Cubo et al. 2019). One interpretation of the HD data is that the time taken for a cell to somatically expand toward the critical toxic threshold in neurons is primarily driven by the length of the largest allele that expands more rapidly and is most likely to reach the threshold first (Kaplan et al. 2007; Hong et al. 2021). In SCA3, it’s possible that because of the lack of a major allele length effect, the two alleles progress more closely toward the toxic neuronal threshold and which of the two alleles crosses the threshold first may be more stochastic. This may also be facilitated by the observation that in most homozygous cases in SCA, the two alleles are relatively close in size differing by ~ 3 repeats (the case here and (Lysenko et al. 2010). In contrast, these differences are typically much larger (~ 8 repeats) in HD (Lee et al. 2012). Moreover, most of the HD homozygotes actually carry one large expanded allele and a reduced penetrance ≤ 39 repeats (Lee et al. 2012; Cubo et al. 2019). It thus remains possible that HD is not truly dominant and that individuals with two relatively closely sized expanded alleles would actually develop symptoms earlier than expected based on the largest allele.

In all of the analyses presented here, we have used the modal allele repeat length observed in the MiSeq distribution in blood DNA as the most objective estimate of the actual inherited allele length of expanded alleles. In many cases, the read length distributions present with a clear mode, a characteristic tail of shorter Taq polymerase slippage products typically extending to ~ N_-5_, and a modest number of expansions typically extending to N_+ 3/4_ (e.g., Fig. S4c, e, j, k, m, s etc.). Such distributions are most commonly observed in samples from individuals sampled at a younger age (< 50 years), and with smaller modal alleles (< 74 repeats). In these cases, we are confident that the modal allele does indeed reflect the inherited progenitor allele length. However, in some individuals with longer alleles and/or sampled at greater ages, the repeat length distributions are more difficult to interpret. For instance, consider the distributions presented in Fig. S4p and S4q. Both have a very similar overall distribution over the same range of repeats, but the mode is one repeat different between them. Considering the distribution in Fig. S4p it is very credible that the mode of 75 represents the inherited progenitor allele and that the high peak at N_+ 1_ (76 repeats), reflects high levels of somatic expansion. Indeed, although we don’t consider it likely, it may even be possible that this allele is so unstable that the actual inherited allele was only 74 repeats and that even the modal peak at 75 repeats represents somatic expansion products. Conversely, it’s possible that the length dependent Taq polymerase slippage is so great that the mode at 75 actually represents primarily N_-1_ slippage products from a true inherited allele length of 76. Similar arguments can be made for the distribution presented in Fig. S4q, with plausible inherited progenitor allele lengths from 74 to 76 repeats. Adding further weight to these concerns is the observation that in one participant the mode observed in the buccal swab DNA was one repeat larger than that observed in blood (Fig. 2i). We believe this represents an example where the degree of somatic expansion in the buccal cells has been so great that the majority of cells have gained at least one repeat. Similarly, a recent inter-tissue comparison revealed modal allele lengths one repeat shorter in the cerebellum than in blood and/or other brain regions (Kacher et al. 2024). Unfortunately, we currently have no objective method to derive the true inherited allele length or provide an alternative estimated progenitor allele length. This inability to objectively define the progenitor allele is the primary limitation of our study, as it will have been in all previous studies of expanded *ATXN3* repeat alleles. Similarly, using this approach we are also not able to quantify somatic contractions as, if they exist, they remain hidden in the tail of PCR slippage errors. A critical imperative for future SCA3 studies is therefore to employ PCR-free long read sequencing approaches (e.g., (Hafford-Tear et al. 2019), or implement single molecule barcoding approaches to correct for PCR slippage (Woerner et al. 2021), in order to potentially identify the progenitor allele and quantify contractions.

Here, we have established the utility of a high-throughput ultra-deep MiSeq sequencing assay to genotype the *ATXN3* repeat, expansions of which cause SCA3. In addition to accurately revealing repeat length, the assay also reveals the exact structure of the complex polyglutamine encoding repeat, phases the rs12895357 SNV and allows for highly sensitive quantification of somatic expansion. Our application of this assay to the Azorean SCA3 cohort has already revealed the limitations of the traditional fragment length analysis approach and yielded new insights into the key age- and tissue-dependence, and apparent modulatory effect of the rs12895357 SNV, on somatic expansion. These observations pave the way for the application of this technology to larger more diverse SCA3 cohorts to augment ongoing efforts to define the *cis*- and *trans*-acting modifiers of genetic instability and clinical phenotypes as therapeutic targets in SCA3.

## Electronic supplementary material

Below is the link to the electronic supplementary material.

Supplementary Material 1

## Data Availability

All data produced in the present study are available upon reasonable request to the authors.
