# Supplementary material for: Age-dependent somatic expansion of the ATXN3 CAG repeat in the blood and buccal swab DNA of individuals with spinocerebellar ataxia type 3/Machado-Joseph disease
Source: Hum Genet. 2024 Oct 8;143(11):1363–78. doi: 10.1007/s00439-024-02698-7 (PMC11522074; doi:10.1007/s00439-024-02698-7)
Supplement: Supplementary file 1 — Supplementary Material 1 [file 439_2024_2698_MOESM1_ESM.pdf]

Age-dependent somatic expansion of the *ATXN3* CAG repeat in the blood and buccal swab DNA of individuals with spinocerebellar ataxia type 3/Machado-Joseph disease

Ahmed M. Sidky,[1,2,3*] Ana Rosa Vieira Melo,[4,5] Teresa T. Kay,[6] Mafalda Raposo,[7] Manuela Lima[4,5] and Darren G. Monckton[1+]

Supplementary information

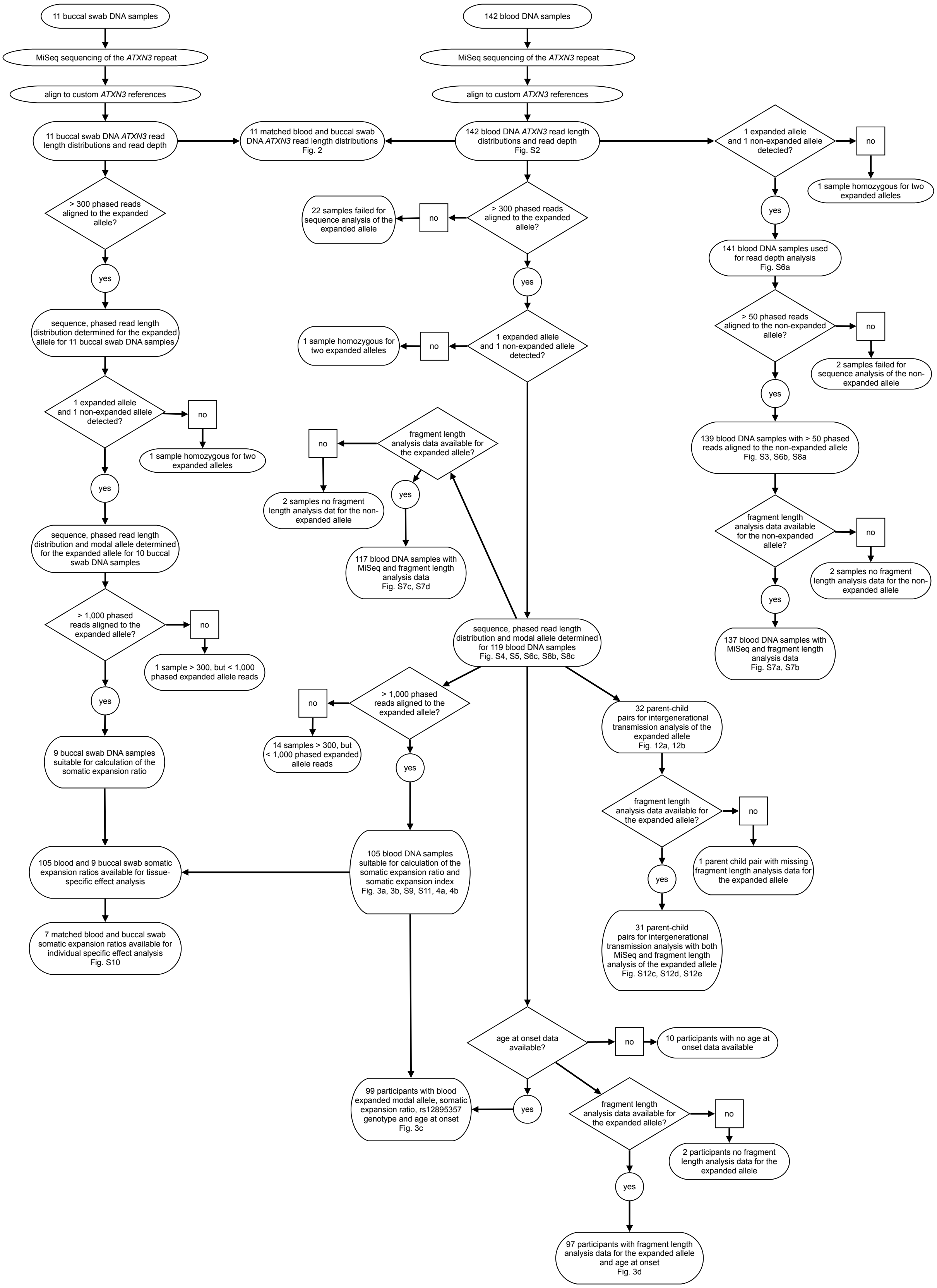

Fig. S1 Samples and data analysis. The flow chart illustrates the samples available, the processing pipeline, and the availability of data-points for relevant analyses and figures.

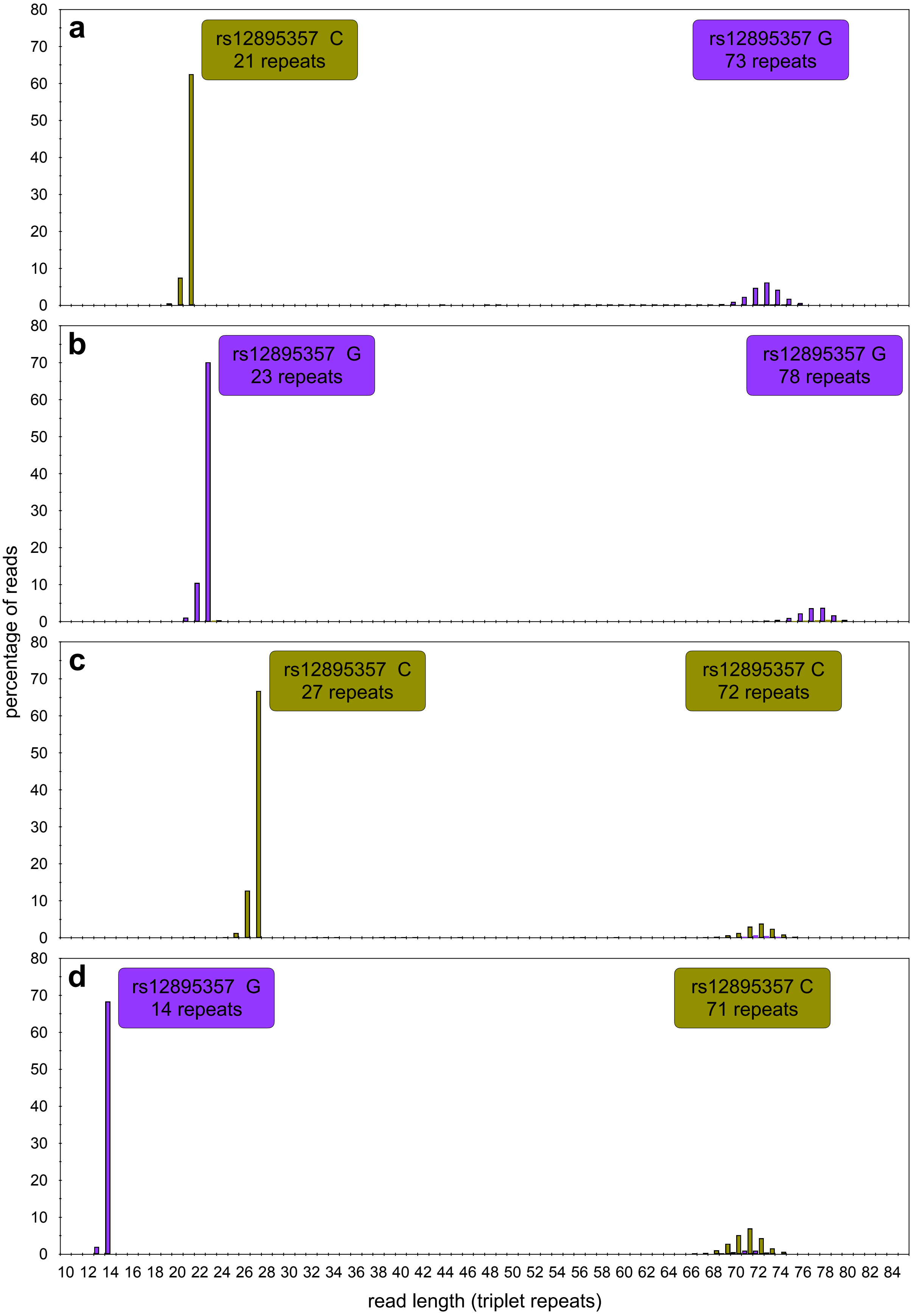

Fig. S2 *ATXN3* triplet repeat rs12895357-specific read-length distributions. The histograms show the read-length distributions for four participants representing all four possible combinations of rs12895357 and expanded and non-expanded *ATXN3* triplet repeat alleles (**a** – **d**). MiSeq reads were aligned against references containing a variable number of CAG repeats and either the rs12895357 C-allele (asparagus) or G-allele (purple). The two read length distributions allow the clear phasing of each triplet repeat allele to its cognate rs12895357 allele.

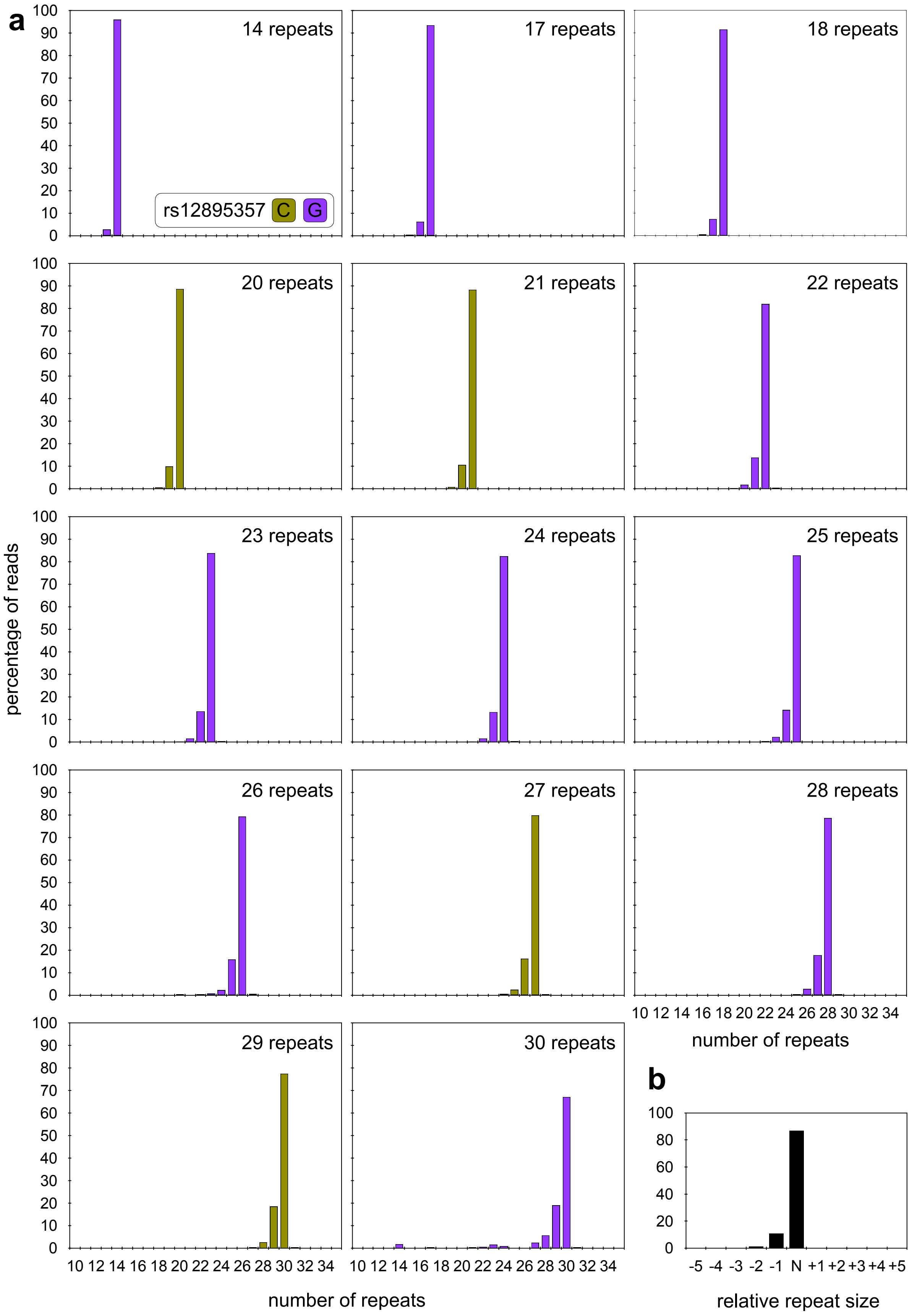

Fig. S3 *ATXN3* triplet repeat phased read-length distributions for non-expanded alleles. **a**) The histograms show representative phased read-length distributions for the non-expanded *ATXN3* allele from participants covering the full range of such alleles. MiSeq reads were aligned against references containing a variable number of CAG repeats and either the rs12895357 C-allele (asparagus) or G-allele (purple). **b)** The histogram shows the averaged normalised phased read length distribution for the non-expanded *ATXN3* alleles (n = 139). The major modal allele is defined as N, and with reads smaller than the mode indicated as N-1, N-2, N-3 *etc.,* and reads larger than the mode indicated as N+1, N+2, *etc.*

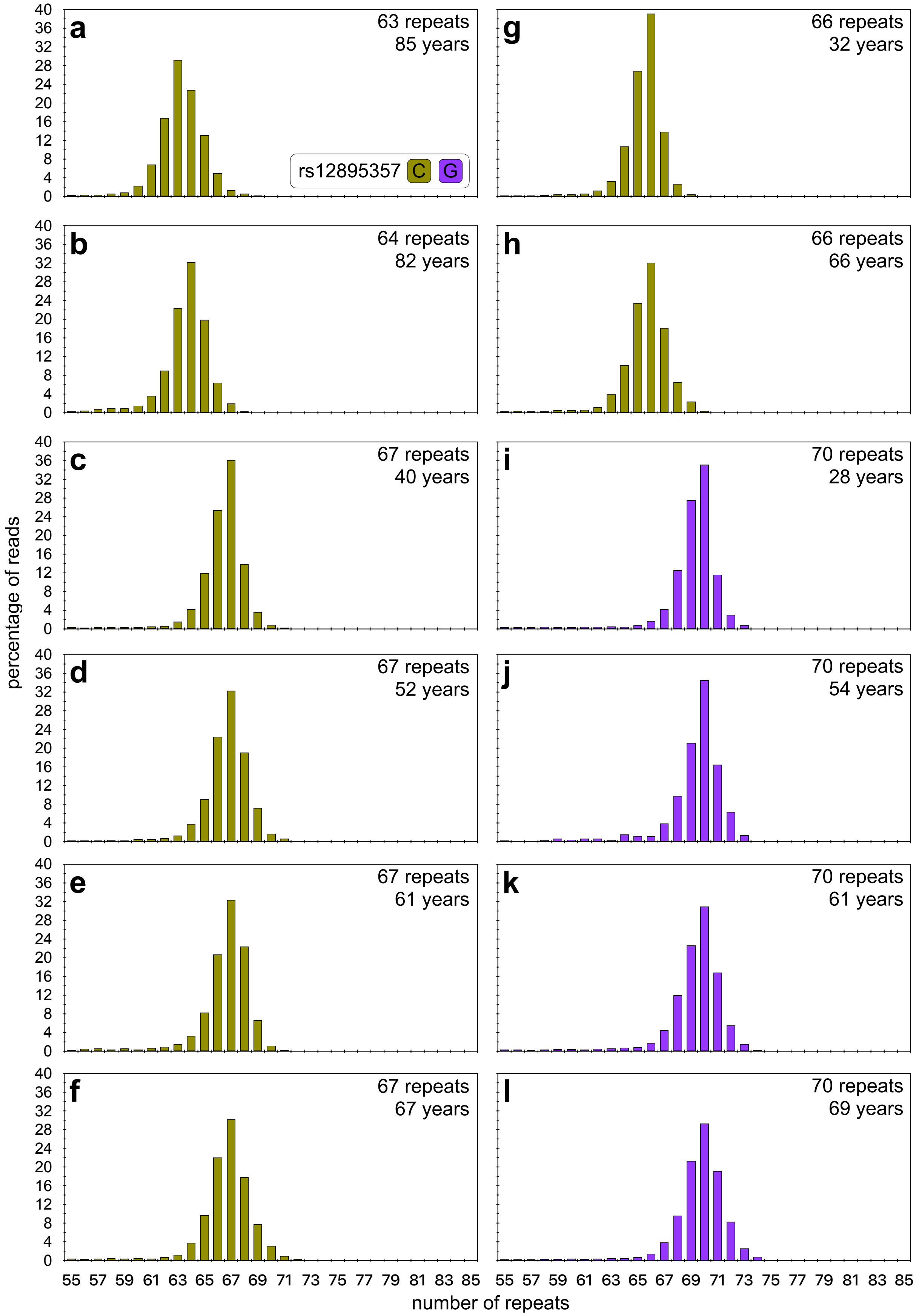
a
63 repeats
85 years
rs12895357
C
G
b
64 repeats
82 years
c
67 repeats
40 years
d
67 repeats
52 years
e
67 repeats
61 years
f
67 repeats
67 years
g
66 repeats
32 years
h
66 repeats
66 years
i
70 repeats
28 years
j
70 repeats
54 years
k
70 repeats
61 years
l
70 repeats
69 years
percentage of reads
number of repeats

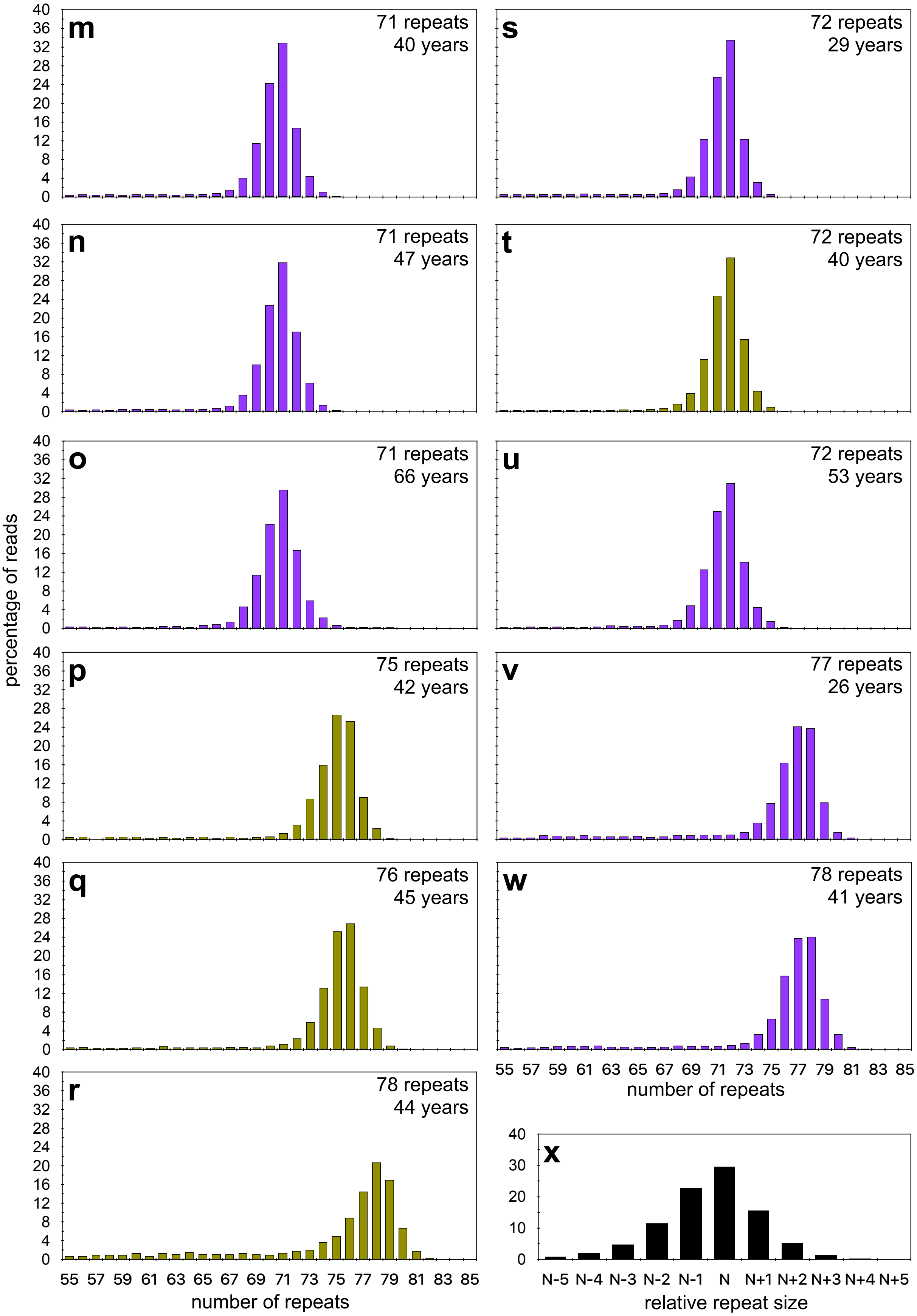

Fig. S4 *ATXN3* triplet repeat phased read-length distributions for expanded alleles. **a** – **w**) The histograms show representative phased read-length distributions for the expanded *ATXN3* allele from participants covering the full range of such alleles. MiSeq reads were aligned against references containing a variable number of CAG repeats and either the rs12895357 C-allele (asparagus) or G-allele (purple). **x**) The histogram shows the average normalised phased read length distribution for expanded *ATXN3* alleles (*n* = 105). The major modal allele is defined as N, and with reads smaller than the mode indicated as N-1, N-2, N-3 *etc.*, and reads larger than the mode indicated as N+1, N+2, N+3 *etc.*

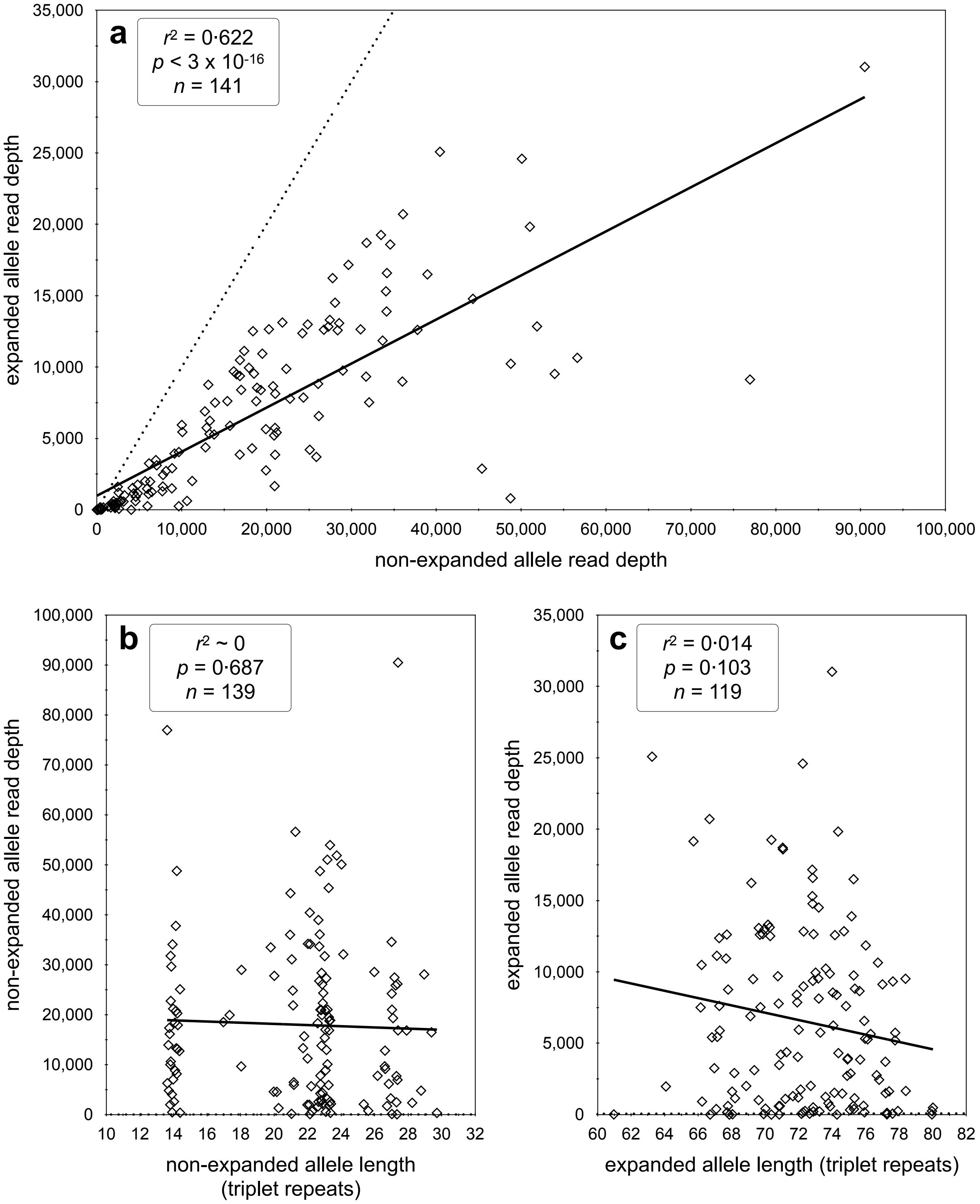

Fig. S5 Read depth analysis of the MiSeq sequencing of the *ATXN3* repeat. **a**) The scatterplot shows the total MiSeq read depth of the non-expanded versus expanded *ATXN3* allele. The expected 1:1 fit is also indicated with a dashed line. **b**) The scatterplot shows the MiSeq phased read depth of the non-expanded allele versus the number of triplet repeats in the non-expanded *ATXN3* allele. **c**) The scatterplot shows the MiSeq phased read depth of the expanded allele versus the number of triplet repeats in the expanded *ATXN3* allele. For (**b**, **c**) the line of best fit (dark black line), adjusted coefficient of correlation squared (r^2), *p*-value (*p*) and sample size (*n*) are indicated. Note, that to allow visualisation of overlapping points, random jitter (up to +/- 0.4 repeats) has been applied to the allele length data points.

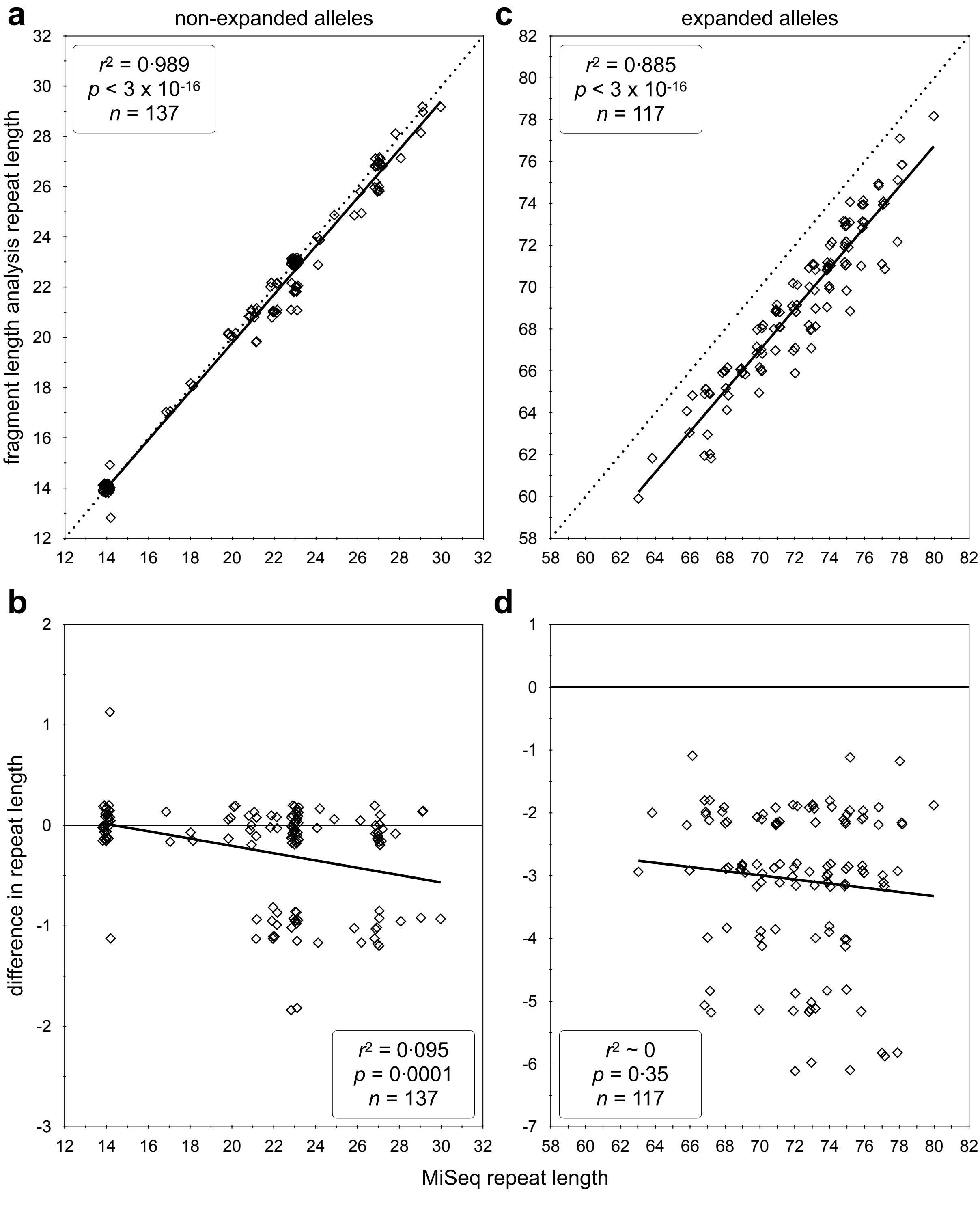

Fig. S6 Modal allele length comparisons between *ATXN3* triplet repeat length determined by MiSeq and fragment length analysis. The scatterplots show MiSeq modal allele length plotted against absolute modal allele length as determined by fragment length analysis (**a**, **c)** or against the difference in modal allele length between MiSeq and fragment length analysis (**b**, **d**) for non-expanded alleles (**a**, **b**) and expanded alleles (**c**, **d**). In each case the line of best fit (dark black line), adjusted coefficient of correlation squared (r^2), *p*-value (*p*) and sample size (*n*) are indicated. For (**a**, **c**) the expected 1:1 fit is also indicated with a dashed line. Note, that to allow visualisation of overlapping points, random jitter (up to +/- 0.4 repeats) has been applied to all data points.

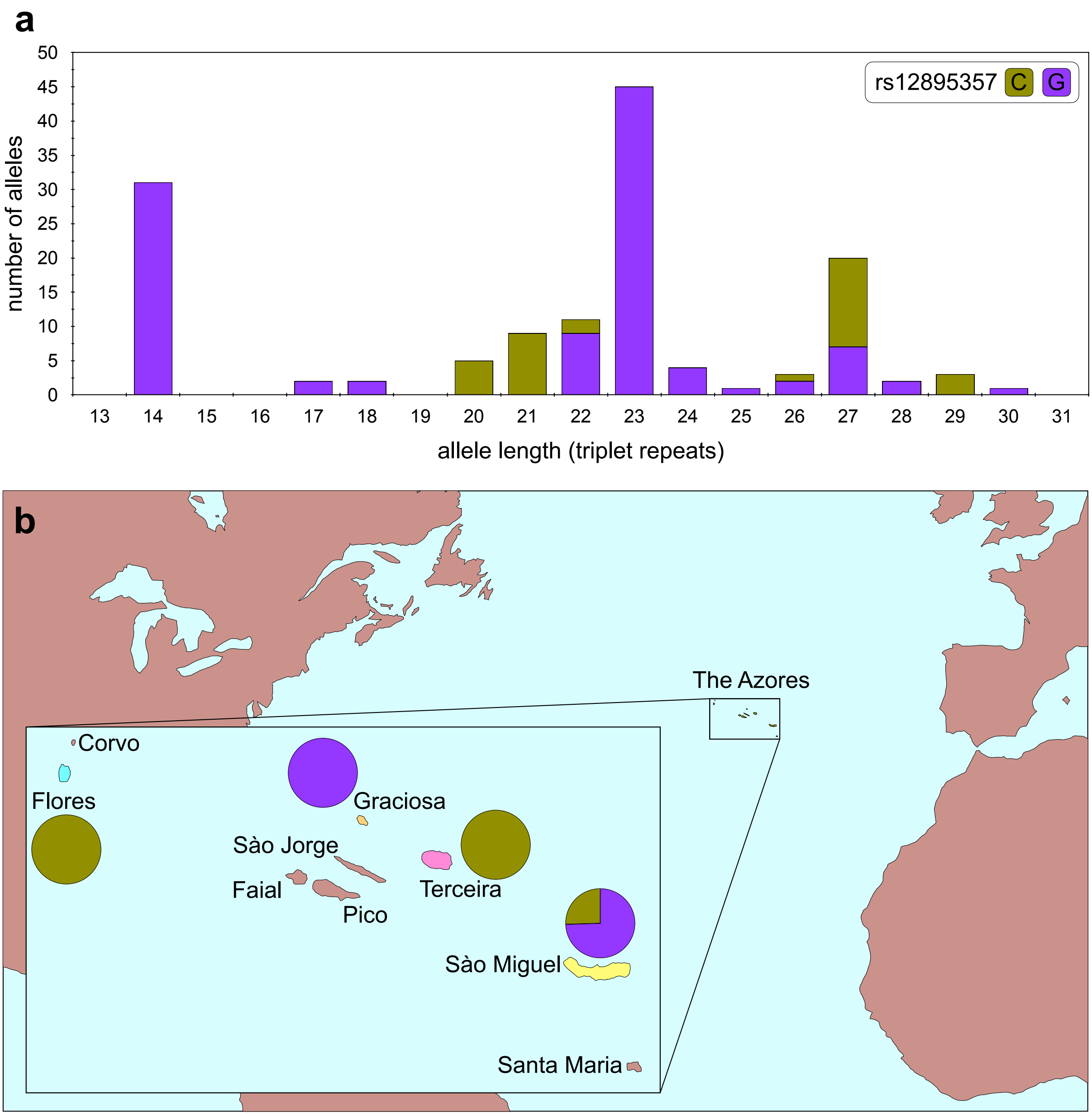

c

rs12895357	island																									
	Flores													Graciosa			Sào Miguel								Terceira	
	F1	F2	F3	F4	F5	F6	F8	F10	F11	F13	F14	F18	F19	F20	G1	S1	S2	S3	S4	S7	S8	S11	S12	S17	S18	T1
G	0	0	0	0	0	0	0	0	0	0	0	0	0	0	12	0	16	3	13	1	3	0	0	3	3	0
C	15	6	2	4	3	1	2	5	0	1	2	2	1	2	0	11	0	0	0	0	0	1	3	0	0	18

Fig. S7 rs12895357 genotypes. **a**) The histogram shows rs12895357 *ATXN3* repeat length associations for the non-expanded alleles. **b**) The map and pie charts show the geographical distribution for the rs12895357 alleles linked to expanded *ATXN3* repeat alleles in the Azores. **c**) The table shows the geographical and familial (F1, F2, G1, S1 *etc.*) distribution for the rs12895357 alleles linked to expanded *ATXN3* repeat alleles in the Azores where each table entry indicates the number of family members with the specific genotype assayed.

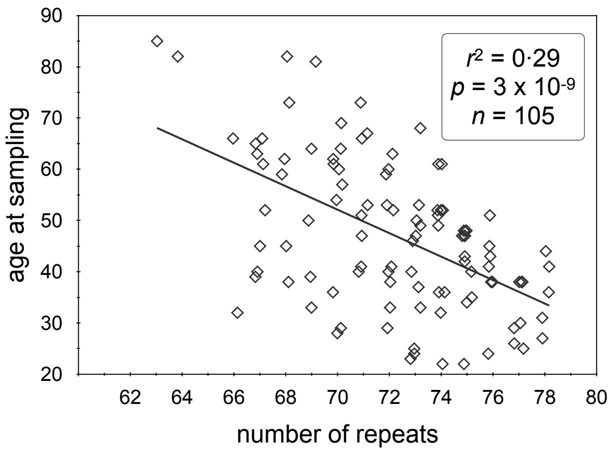

Fig. S8 Age at sampling bias in SCA3. The scatterplot shows the inverse association between age at sampling of SCA3 and *ATXN3* expanded allele repeat length in the Azorean SCA3 cohort. The line of best fit (black line), adjusted coefficient of correlation squared (r^2), *p*-value (*p*), and sample size (*n*) are indicated. Note, that to allow visualisation of overlapping points, random jitter (up to +/- 0.4 repeats) has been applied to the number of repeats.

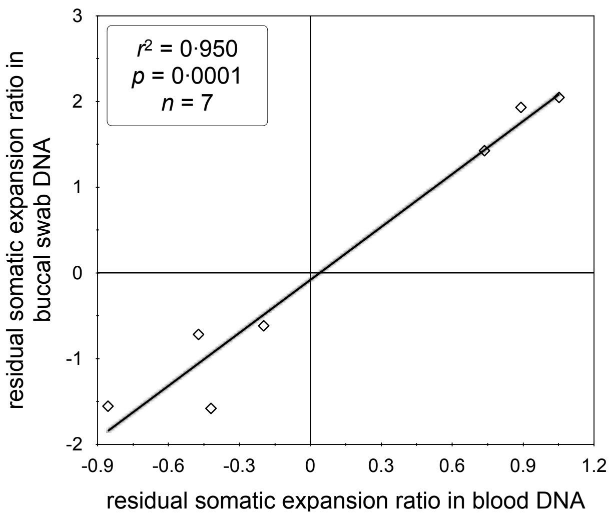

Fig. S9 Shared individual specific somatic ratios between blood and buccal swab DNA samples. The scatterplot shows the positive association between residual variation in somatic expansion ratio corrected for age, repeat length, rs12895357 genotype and tissue in matched individuals for blood and buccal swab DNA. The line of best fit (black line), adjusted coefficient of correlation squared (r^2), *p*-value (*p*), and sample size (*n*) are indicated.

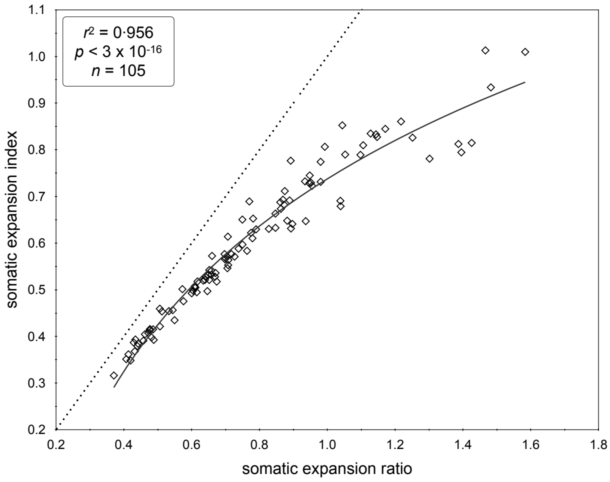

Fig. S10 The somatic expansion ratio and the somatic expansion index of the *ATXN3* expanded allele. The scatterplot shows the positive association between the somatic expansion ratio and the somatic expansion index in blood DNA. The logarithmic line of best fit (black line), adjusted coefficient of correlation squared (r^2), p-value (p), and sample size (n) are indicated. The 1:1 fit is also indicated with a dashed line.

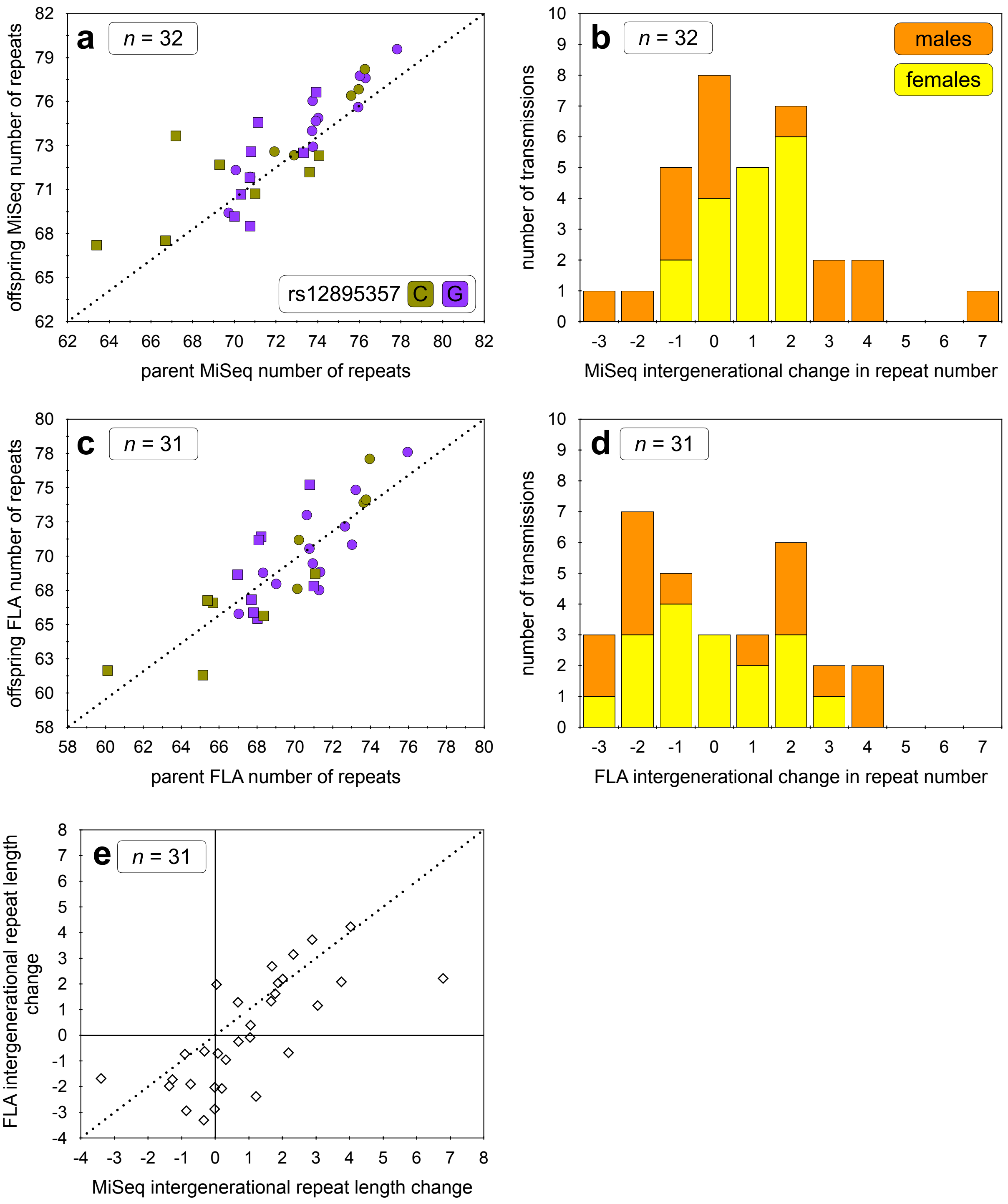

Fig. S11 Intergenerational transmissions in SCA3. **a**) The scatterplot shows the association between *ATXN3* expanded modal allele repeat length in parent and offspring in the Azorean SCA3 cohort as determined using the MiSeq analysis. The expected 1:1 line of no change is indicated (dashed line). **b**) The histogram shows the relative difference in *ATXN3* modal expanded allele repeat length between parent and offspring in the Azorean SCA3 cohort for both paternal (orange) and maternal (yellow) transmissions as determined using the MiSeq analysis. **c**) The scatterplot shows the association between *ATXN3* expanded allele repeat length in parent and offspring in the Azorean SCA3 cohort as determined using fragment length analysis (FLA). The expected 1:1 line of no change is indicated (dashed line). **d**) The histogram shows the relative difference in *ATXN3* expanded allele repeat length between parent and offspring in the Azorean SCA3 cohort for both paternal (orange) and maternal (yellow) transmissions as determined using fragment length analysis (FLA). **e**) The scatterplot shows the association between *ATXN3* expanded allele intergenerational transmissions as determined using fragment length (FLA) versus MiSeq analysis. The expected 1:1 line of methodological parity is indicated (dashed line). Note, that to allow visualisation of overlapping points, random jitter (up to +/- 0.4 repeats) has been applied to the number of repeats in scatterplots **a**, **c** and **e**.
